# *Cnot3* is required for male germ cell development and spermatogonial stem cell maintenance

**DOI:** 10.1242/dev.204557

**Published:** 2025-08-15

**Authors:** Qing Chen, Safia Malki, Xiaojiang Xu, Jiajia Wang, Brian Bennett, Xiaofeng Zheng, Brad L. Lackford, Oleksandr Kirsanov, Christopher B. Geyer, Guang Hu

**Affiliations:** ^1^ Epigenetics and Stem Cell Biology Laboratory, National Institute of Environmental Health Sciences, Research Triangle Park, NC 27709, USA; ^2^ Integrative Bioinformatics Support Group, National Institute of Environmental Health Sciences, Research Triangle Park, NC 27709, USA; ^3^ School of Veterinary Medicine, Hunan Agricultural University, Changsha, Hunan 410127, China; ^4^ Department of Anatomy & Cell Biology, Brody School of Medicine at East Carolina University, Greenville, NC 27834, USA; ^5^ East Carolina Diabetes and Obesity Institute at East Carolina University, Greenville, NC 27858-4353, USA

**Keywords:** Spermatogonial stem cells, Maintenance, Spermatogenesis, scRNA-seq, Glutathione redox pathway, Mouse

## Abstract

The foundation of spermatogenesis and lifelong fertility is provided by spermatogonial stem cells (SSCs). SSCs divide asymmetrically to either self-renew or produce undifferentiated progenitors. However, regulatory mechanisms governing SSC maintenance are poorly understood. Here, we show that the CCR4-NOT mRNA deadenylase complex subunit CNOT3 is essential for sustaining spermatogonial populations in mice. Its deletion in adult germ cells resulted in germ cell loss and infertility, and its deletion in spermatogonia in the developing testis resulted in SSC depletion and compromised spermatogenesis. Consistent with the *in vivo* results, deletion of *Cnot3* in cultured SSCs caused a reduction in cell proliferation and viability, and downregulation of SSC markers. Mechanistically, *Cnot3* deletion led to the de-repression of transcripts encoding factors involved in spermatogonial differentiation, including those in the glutathione redox pathway that are crucial for SSC maintenance. Together, our study reveals that CNOT3 – likely via the CCR4-NOT complex – promotes the degradation of transcripts encoding differentiation factors to maintain the SSCs in the stem cell state, highlighting the importance of CCR4-NOT-mediated post-transcriptional gene regulation in SSCs and male germ cell development.

## Teaser

**Summary:** Conditional deletion of *Cnot3* leads to total germ cell loss and male infertility. CNOT3 represses transcripts encoding factors involved in spermatogonial differentiation and protects SSC maintenance via the GSH/redox pathway.

## INTRODUCTION

Spermatogenesis is a stem cell-based developmental program that produces large numbers of haploid gametes every day to sustain lifelong male fertility. Originating in the fetal testis, prospermatogonia (also called gonocytes), transition after birth into spermatogonia, a subset of which are spermatogonial stem cells (SSCs) (de Rooij, 2001). These SSCs balance their divisions during steady-state spermatogenesis to both replenish their population (via self-renewal) and produce undifferentiated progenitors that proliferate before differentiating (Oatley and Brinster, 2006). Differentiation occurs in response to retinoic acid (RA), which ensures an irreversible commitment to ultimately enter meiosis (Gewiss et al., 2021; Griswold, 2016; Koubova et al., 2006; Velte et al., 2019). After five divisions, these differentiating spermatogonia become preleptotene spermatocytes, which divide one final time before entering meiosis. Following the lengthy meiotic program, spermatocytes divide twice to form haploid round spermatids that undergo the morphogenetic program of spermiogenesis to become sperm (de Kretser et al., 1998; de Rooij and Russell, 2000).

The maintenance of the SSC pool is orchestrated by both transcriptional and post-transcriptional mechanisms (Legrand and Hobbs, 2018). While the roles of several RNA-binding proteins have been described during spermatogenesis, only a handful, e.g. NANOS2, DND1 and TRIM71, have been implicated in SSCs (Du et al., 2020; Legrand and Hobbs, 2018; Niimi et al., 2019; Zhou et al., 2015).

We previously reported that the CNOT1, CNOT2 and CNOT3 subunits of the CCR4-NOT complex maintain the pluripotent state in mouse embryonic stem cells by preventing their differentiation into extra-embryonic lineages (Zheng et al., 2012). Deletion of *Cnot3* in embryonic stem cells reveals a role in promoting the deadenylation and subsequent degradation of mRNAs encoding differentiation factors, revealing a requirement for mRNA decay in the maintenance of the pluripotent state (Hu et al., 2009; Zheng et al., 2012). In mice, deletion of *Cnot3* causes embryonic lethality at the blastocyst stage due to loss of the inner cell mass (Neely et al., 2010; Zheng et al., 2016).

In this study, we generated male germ cell-specific *Cnot3* knockout (KO) mice and identified a crucial role for CNOT3 in maintaining the SSC reserve. We show that, in the developing testis of the *Cnot3* KO animals, SSC and progenitor populations in the spermatogonia were depleted, leading to loss of the germline and male sterility. Furthermore, *Cnot3* deletion resulted in the de-repression of transcripts encoding factors involved in SSC differentiation, including those in the glutathione (GSH) redox pathway that are essential for SSC maintenance. Together, our results indicate that CNOT3-mediated mRNA decay serves as an essential post-transcriptional regulatory mechanism in SSC maintenance.

## RESULTS

### *Cnot3* deletion in adult mice results in germ cell loss and male infertility

As a first step to explore the role of CNOT3 during mouse spermatogenesis, we assessed *Cnot3* transcript levels in published single-cell RNA sequencing (scRNA-seq) data (Green et al., 2018). We found that *Cnot3* mRNAs are abundant in spermatogonia, but later decrease in spermatocytes, round spermatids and elongating spermatids (Fig. S1A). At the protein level, immunostaining results showed that CNOT3 is detectable in ZBTB16 (also known as PLZF)-positive cells located along the basement membrane of seminiferous tubules in adult mouse testes (Fig. S1B,C). ZBTB16-positive cells in the testes are undifferentiated spermatogonia, comprising both the SSC and progenitor populations (Buaas et al., 2004; Costoya et al., 2004; Hobbs et al., 2010). Thus, we posited a role for CNOT3 in undifferentiated spermatogonia.

**Fig. 1.**
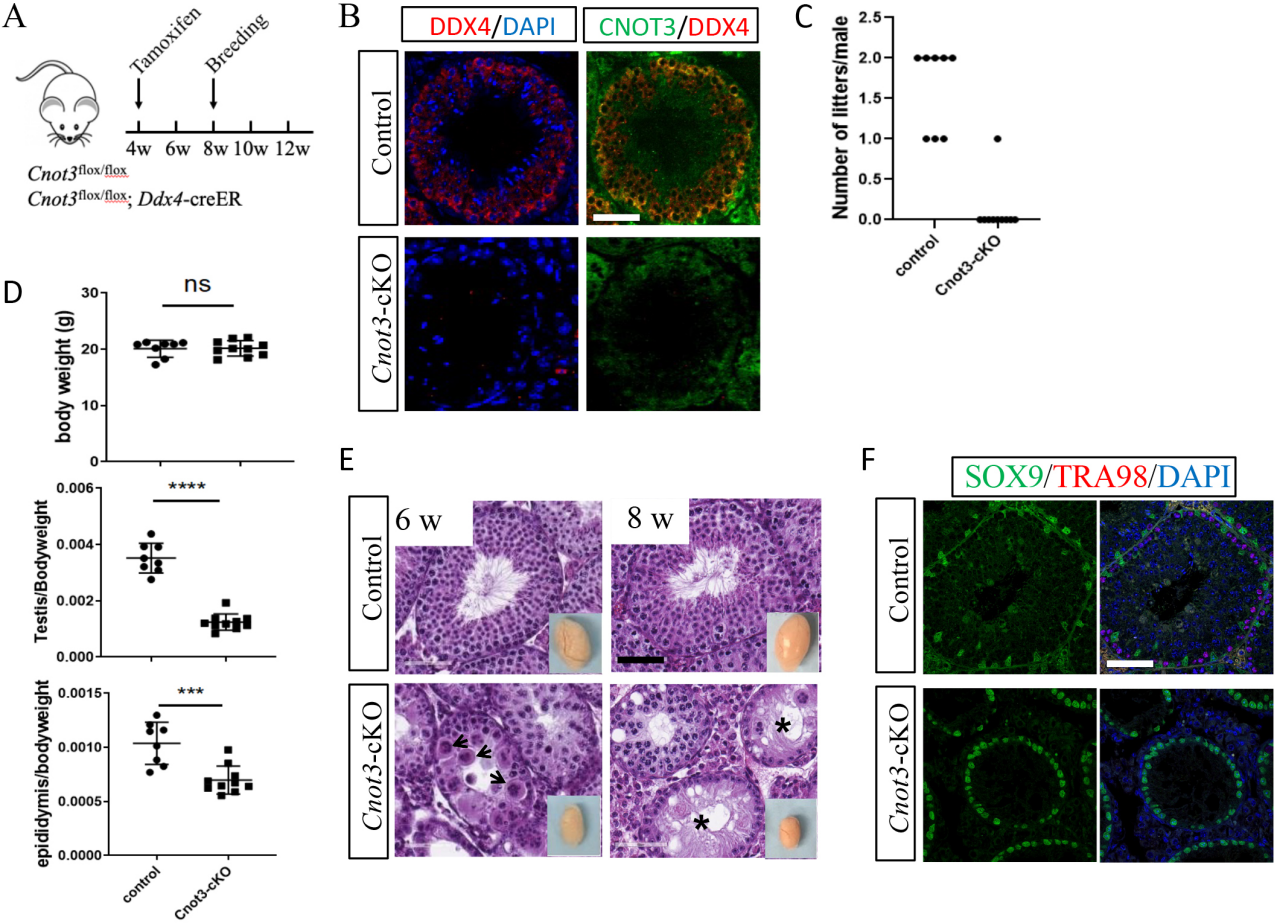
***Cnot3* deletion results in germ cell loss and male infertility.** (A) Schematic of conditional deletion of *Cnot3* in male germ cell upon 4-OHT (tamoxifen) administration. (B) Merged channels of single confocal sections of 8-week-old control and *Cnot3*-cKO testis showing CNOT3 (green) and DDX4 (red) expression with DAPI (blue). (C) Quantification of live litters obtained from WT females mated with control and *Cnot3*-cKO males. *n*=8 for control; *n*=10 for *Cnot3*-cKO. (D) Quantification of body weight in grams, testis to body weight ratio and epididymis to body ratio weight ratio of 8-week-old control and *Cnot3*-cKO males. ***P<0.001, ****P<0.0001 (two-tailed, unpaired Student's *t*-test). ns, not significant (*P*>0.05). Data are mean±s.e.m. *n*=8 for control; *n*=10 for *Cnot3*-cKO. (E) Hematoxylin and Eosin-stained sections of testes of 6- and 8-week-old control and *Cnot3*-cKO males. Insets show whole testes. Arrowheads and asterisks indicate multinucleated degenerating cells and seminiferous tubules lacking germ cells, respectively. (F) Merged channels of single confocal sections of 8-week-old control and *Cnot3*-cKO testis showing SOX9 (green) and TRA98 (red) expression with DAPI (blue). Images are representative of at least three independent biological samples. w, weeks of age. Scale bars: 60 µm.

To elucidate the function of CNOT3 in spermatogonia, we generated a conditional KO mouse model in which *Cnot3* was specifically deleted in germ cells by the tamoxifen-inducible *Ddx4*-Cre transgene (*Cnot3*^flox/flox^; *Ddx4*-creER) (Zheng et al., 2016), hereafter referred to as *Cnot3*-cKO. To induce Cre expression, we dosed mice with tamoxifen (4-OHT) at 4 weeks of age and examined the fertility of adult (8-week-old) *Cnot3*-cKO males (Fig. 1A). We first confirmed *Cnot3* deletion by immunostaining (Fig. 1B). We then performed a breeding study for 6 weeks by pairing 8-week-old wild-type (WT) control and *Cnot3*-cKO males with WT females (Fig. 1A). Whereas control males produced one or two litters, all but one *Cnot3*-cKO male were sterile and had no litter (Fig. 1C). We next euthanized additional 4-OHT-treated *Cnot3*-cKO males at 6 and 8 weeks old and recorded body, testis and epididymis weights. Although *Cnot3*-cKO body weights did not differ from controls, we observed a significant reduction in the ratios of testis and epididymis to body weights (Fig. 1D). Histological analyses revealed *Cnot3*-cKO males had smaller testes (Fig. 1E), with multinucleated degenerating cells in testes of 6-week-old *Cnot3*-cKO mice and an absence of germ cells in seminiferous tubules of 8-week-old mice (Fig. 1E). The absence of germ cells was confirmed by immunostaining, in which the pan germ cell-specific marker TRA98 was nearly undetectable in *Cnot3*-cKO testes (Fig. 1F). In contrast, the Sertoli cell-specific marker SOX9 showed similar expression pattern in WT and *Cnot3*-cKO testis, indicating that Sertoli cell numbers were not altered in *Cnot3*-cKO mice (Fig. 1F). The infertility and total loss of germ cells in *Cnot3*-cKO testes suggested a loss of SSCs and thereby a potential role of *Cnot3* in SSC maintenance.

### *Cnot3* deletion in neonatal mice results in spermatogonia pool depletion

**Fig. 2.**
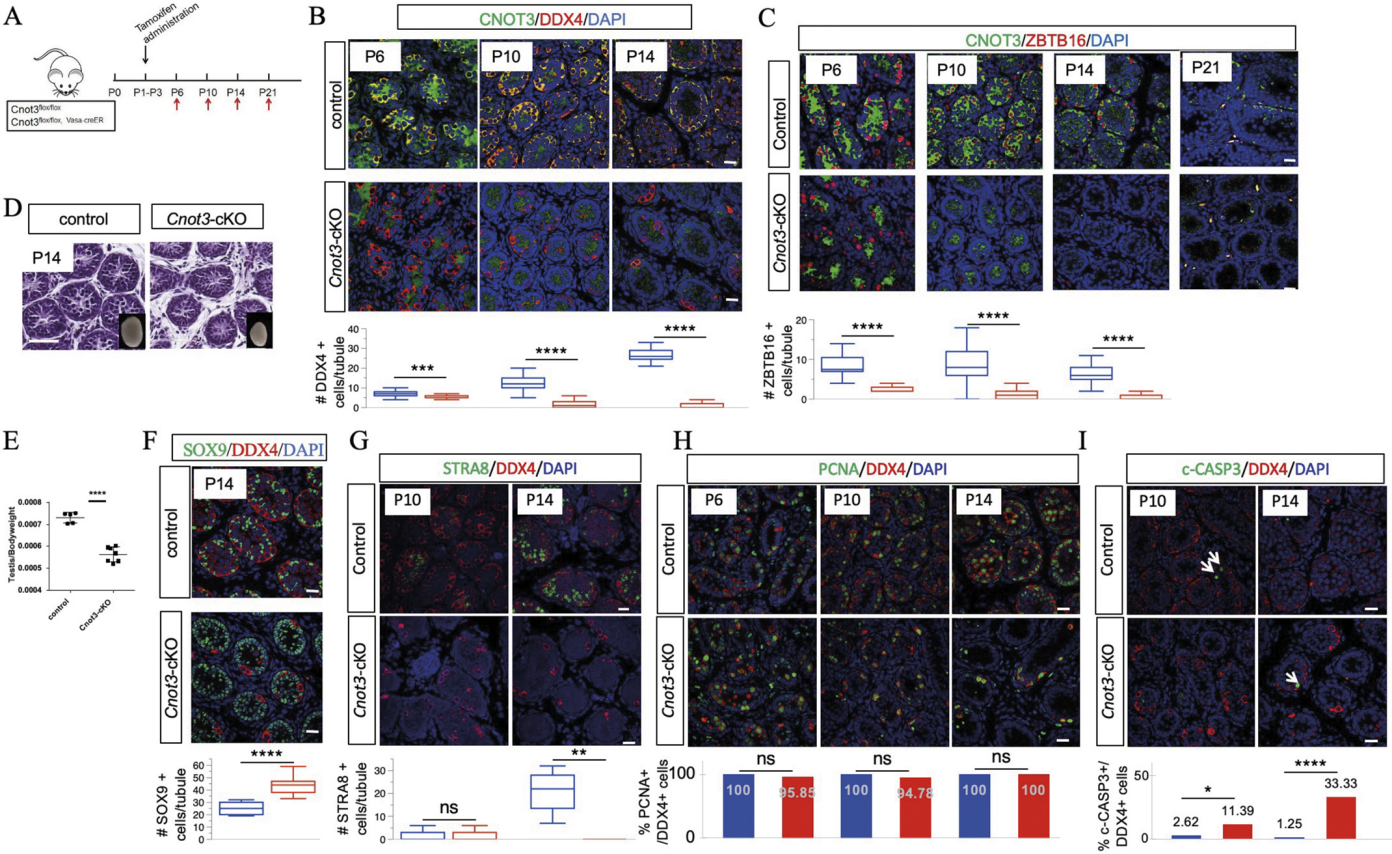
***Cnot3* deletion causes depletion of ZBTB16^+^ spermatogonia and decrease of SSC maintenance.** (A) Experimental scheme to achieve and analyze the deletion of *Cnot3* in neonatal testis. Red arrows indicate sample collection time points. (B) Merged channels of single confocal sections of P6, P10 and P14 control and *Cnot3*-cKO testis showing CNOT3 (green) and DDX4 (red) expression with DAPI (blue). The number of positively labeled cells per tubule from at least three independent biological measurements are shown. ***P<0.001, ****P<0.0001 (two-tailed Kolmogorov–Smirnov test). (C) Merged channels of single confocal sections of P6, P10, P14 and P21 control and *Cnot3*-cKO testis showing CNOT3 (green) and ZBTB16 (red) expressing cells with DAPI (blue). The number of positively labeled cells per tubule from at least three independent biological measurements are shown. ****P<0.0001 (two-tailed Kolmogorov–Smirnov test). (D) Hematoxylin and Eosin-stained sections of testes of P14 WT and *Cnot3*-cKO males. Insets show whole testes. Images are representative of 5 control and 7 *Cnot3*-cKO samples. (E) Quantification of testis to body weight ratio of P14 control and *Cnot3*-cKO males. ***P<0.0001 (two-tailed, unpaired Student's *t*-test). Data are mean±s.e.m. *n*=5 for control; *n*=7 for *Cnot3*-cKO. (F) Merged channels of single confocal sections of P14 control and *Cnot3*-cKO testis showing SOX9 (green) and DDX4 (red) expressing cells with DAPI (blue). The number of positively labeled cells per tubule from at least three independent biological measurements are shown. ****P<0.0001 (two-tailed Kolmogorov–Smirnov test). (G) Merged channels of single confocal sections of P10 and P14 control and *Cnot3*-cKO testis showing STRA8 (green) and DDX4 (red) expression with DAPI (blue). The number of positively labeled cells per tubule from at least three independent biological measurements are shown. **P<0.01 (two-tailed Kolmogorov–Smirnov test). ns, not significant (*P*>0.05). (H) Merged channels of single confocal sections of P6, P10 and P14 control and *Cnot3*-cKO testis showing PCNA (green) and DDX4 (red) expression with DAPI (blue). The percentage of double-positive cells from at least three independent biological measurements is shown. ns, not significant (*P*>0.05; two-tailed Fisher's test). (I) Merged channels of single confocal sections of P10 and P14 control and *Cnot3*-cKO testis showing c-CASP3 (green) and DDX4 (red) expression with DAPI (blue). Arrows indicate c-CASP3 positive cells. The percentage of double-positive cells from at least three independent biological measurements is shown. *P<0.05, ****P<0.0001 (two-tailed Fisher's test). Scale bars: 20 μm. Box and whisker plots display the five-number summary: minimum, first quartile, median, third quartile and maximum.

During normal development, by postnatal day (P) 3 quiescent prospermatogonia re-enter the cell cycle, migrate to the basement membrane of the testis cords, and transition into spermatogonia (Drumond et al., 2011; Nagano et al., 2000). This foundational population of spermatogonia contains both precursors that will form future adult SSCs, as well as undifferentiated progenitors and differentiating spermatogonia that will initiate the first wave of spermatogenesis (Hermann et al., 2015; Kluin and de Rooij, 1981; Law et al., 2019; Nikolova et al., 1997; Yoshida et al., 2006). Based on this, we examined the role of *Cnot3* in SSCs more specifically by deleting *Cnot3* in this newly formed spermatogonial population in neonatal testes. We dosed *Cnot3*^flox/flox^; *Ddx4-*CreER mice and *Cnot3*^flox/flox^ control littermates with 4-OHT from P1 to P3 and collected testes at P6, P10, P14 and P21 (Fig. 2A, Fig. S2A). At these ages, testes contain the following most advanced germ cell types: P6, spermatogonia; P10, leptotene spermatocytes; P14, pachytene spermatocytes; P21, round spermatids. We first immunostained testes for CNOT3, the pan germ cell marker DDX4 and the Sertoli cell marker SOX9. We observed absence of CNOT3 protein by P6 (Fig. 2B,C). At P14, seminiferous tubules of *Cnot3*-cKO mice contained very few DDX4^+^ germ cells, but numerous SOX9^+^ Sertoli cells (Fig. 2B,F, Fig. S2B). Consistent with this, *Cnot3*-cKO testes appeared smaller by P14, and the histology analysis showed that, although the newly formed seminiferous tubule structures appeared intact, their diameters were decreased and the interstitial space was increased (Fig. 2D). Average testis-to-body weight ratios of these *Cnot3*-cKO P14 testes were significantly reduced compared to control testes (Fig. 2E). These observations indicated that CNOT3 deletion in the spermatogonia of newborn animals causes complete germ cell loss, similar to results in *Cnot3*-cKO adult mice.

To better understand the impact of *Cnot3* deletion on SSCs, we analyzed the presence of ZBTB16^+^ undifferentiated spermatogonia in control and *Cnot3*-cKO mouse testis. The numbers of ZBTB16^+^ spermatogonia in P6 *Cnot3*-cKO testis were reduced (Fig. 2C). ZBTB16^+^ spermatogonia were dramatically reduced at P10 and were lost entirely in P14 and P21 in *Cnot3*-cKO mouse testes (Fig. 2C), indicating that *Cnot3* deletion led to eventual loss of the undifferentiated spermatogonial pool, which includes the foundational adult SSCs. Consistent with this, the RA-responsive germ cell-specific protein STRA8 (activated in response to RA at the initiation of spermatogonial differentiation) was detected in control but not *Cnot3*-cKO mouse testes at P14 (Fig. 2G), indicating an absence of differentiating spermatogonia in the *Cnot3*-cKO due to the loss of undifferentiated spermatogonia. Importantly, in *Cnot3*-cKO testes, the remaining DDX4^+^ germ cells were still positive for the proliferation marker PCNA at P6, P10 and P14 (Fig. 2H), suggesting that the loss of undifferentiated spermatogonia was not merely caused by defects in germ cell proliferation. Finally, we observed increased cleaved (c)-CASP3^+^ cells (indicative of apoptosis) in *Cnot3*-cKO testes at either P10 or P14 (Fig. 2I). Thus, the germ cell loss in cKO testes appears to be mediated by apoptosis. Together, these results strongly suggest an essential role for CNOT3 in the maintenance of the nascent spermatogonial populations in the developing testis.

### *Cnot3* deletion impairs the maintenance of steady-state spermatogenesis

As described above, the spermatogonial pool in the developing testis is heterogeneous and comprises SSCs, undifferentiated progenitors and differentiating spermatogonia. Therefore, we performed scRNA-seq to examine the impact of *Cnot3* deletion on these distinct spermatogonial subpopulations. We used antibodies against the surface markers CD9 and THY1 in a fluorescence-activated cell sorting approach to enrich for germ cells from freshly prepared P8 control and *Cnot3*-cKO testis single-cell suspensions. We performed scRNA-seq using the 10x Genomics platform on 4027 cells and 4212 cells from control and *Cnot3*-cKO testes, respectively.

**Fig. 3.**
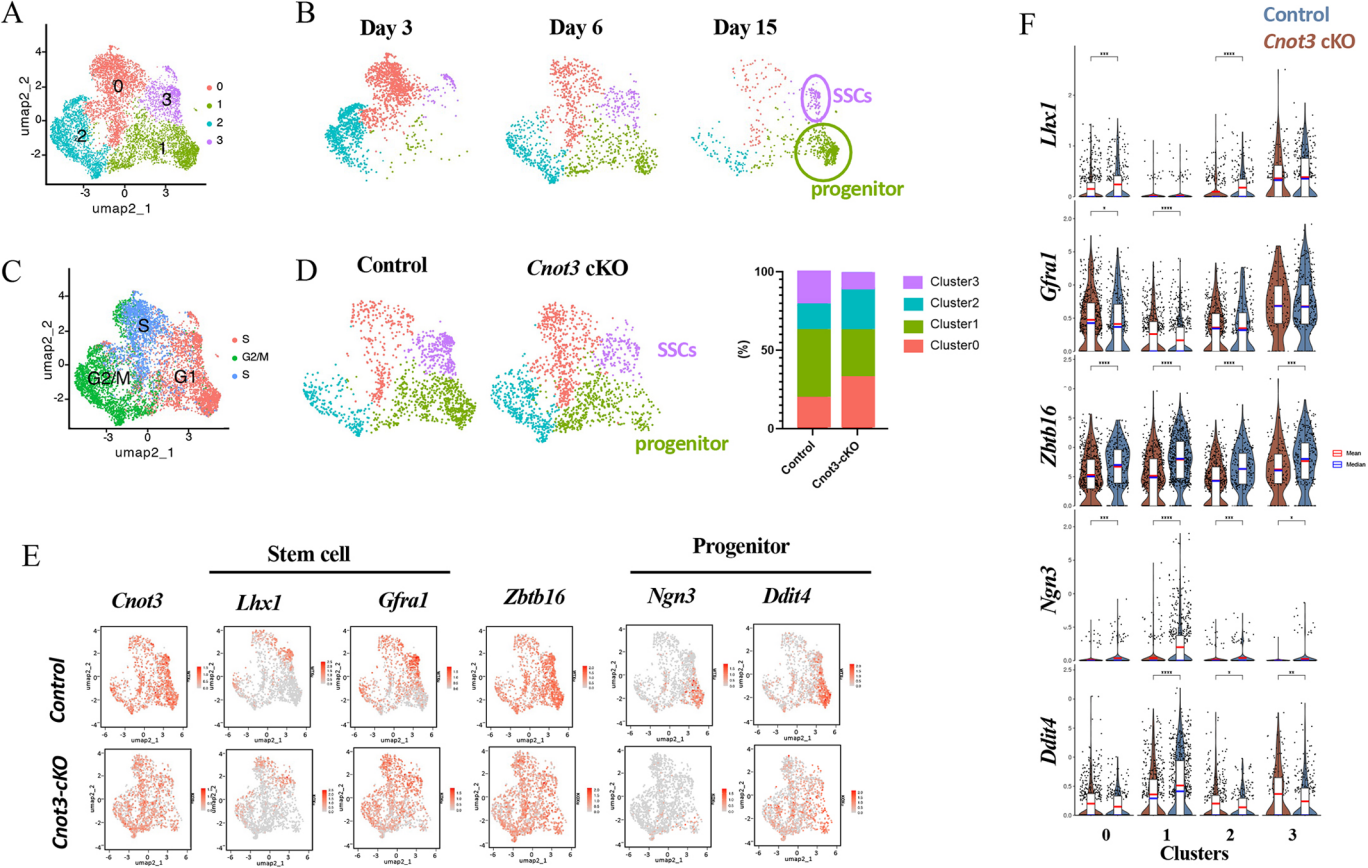
***Cnot3* deletion impairs the maintenance of steady-state spermatogenesis.** (A) Reclustering of the group of cells containing SSCs and progenitor cells from the combination of our data with published datasets from P3, P6, P8, P10 and P15 stages (Law et al., 2019; Grive et al., 2019; Ernst et al., 2019). (B) Reclustering of the group of cells containing SSCs and progenitor cells from published datasets from P3, P6 and P15 stages, respectively (Law et al., 2019; Grive et al., 2019; Ernst et al., 2019). (C) Highlights of cell cycle phases in reclustered group of cells containing SSCs and progenitor cells from the combination of our data with published datasets from P3, P6, P8, P10 and P15 stages (Law et al., 2019; Grive et al., 2019; Ernst et al., 2019). (D) Reclustering and quantification of the groups of cells containing SSCs and progenitor cells from P8 control and *Cnot3*-cKO cells. (E) Highlights of *Cnot3*, stem cell (*Lhx1* and *Gfra1*) and progenitor cell (*Ngn3* and *Ddit4*) markers in reclustered group of cells containing SSCs and progenitor cells from P8 control and *Cnot3-cKO* cells. (F) Expression levels of stem cell (Lhx1, Zbtb16 and Gfra1) and progenitor cell (Ngn3 and Ddit4) markers in reclustered groups of cells containing SSCs and progenitor cells from the combination of our data with published datasets from P3, P6, P8, P10 and P15 stages (Law et al., 2019; Grive et al., 2019; Ernst et al, 2019).

To identify the germ cell populations in these samples, we combined our data with published datasets from P3, P6, P10 and P15 control testes (Ernst et al., 2019; Grive et al., 2019; Law et al., 2019). In the combined dataset, we first identified all the germ cells based on expression of *Dazl* and *Ddx4* (germ cell markers) (Fig. S3). We then used unsupervised clustering and uniform manifold approximation and projection (UMAP) plots to separate the germ cells into 13 distinct cell populations (Fig. S4A,B). Based on marker gene expression and the developmental time, we separated germ cell clusters into four groups: SSCs, progenitor spermatogonia, differentiating spermatogonia and preleptotene spermatocytes (Fig. 3, Fig. S4C). Clusters 0 and 2 first appeared at P3 and become less abundant thereafter (Fig. S4A-D). Germ cells in these clusters also contained high mRNA levels for *Lhx1*, *Gfra1* and *Zbtb16*, which are enriched in SSCs (Grisanti et al., 2009; Niedenberger et al., 2015; Oatley et al., 2006) (Fig. S4C). It is noteworthy that we previously reported that *Zbtb16* is expressed in nearly all spermatogonia (including undifferentiated and differentiating), during the first wave of spermatogenesis (Fig. S4C) (Niedenberger et al., 2015). Based on these results, these clusters were designated as SSCs (Fig. S4A-C). Clusters 1 and 3 became apparent at P6, and included the established undifferentiated progenitor markers *Neurog3* (also termed *Ngn3*), *Ddit4* and *Sox3* (Fig. S4A-C), and thus were classified as undifferentiated progenitor spermatogonia (Hobbs et al., 2010; Raverot et al., 2005; Yoshida et al., 2004). Clusters 1, 5 and 10 also appeared at P6 and were more abundant at P15. These clusters were enriched for the differentiating spermatogonia markers *Stra8* and *Kit* (Fig. S4A-C) and were thus classified as differentiating spermatogonia (Koubova et al., 2006; Schrans-Stassen et al., 1999). Clusters 6, 7, 8, 9 and 11 appeared at P15 and included the meiotic marker *Meioc* (Fig. S4C) and were thus classified as spermatocytes (Abby et al., 2016; Soh et al., 2017).

Since CNOT3 likely plays a crucial role in spermatogonia, we focused on germ cell clusters 0, 2, 3 and 4, which contained both SSCs and undifferentiated progenitors. We re-analyzed these cells and generated a new UMAP to separate cells into four new clusters (Fig. 3A). In the new UMAP, cells from clusters 0 and 2 were predominantly found in P3 testis samples and gradually disappeared along the development toward P15 (Fig. 3A,B), suggesting these are spermatogonia undergoing the first wave of spermatogenesis. In addition, the cell cycle status of these cells was S/G2/M (Fig. 3C, Fig. S4D), which is concordant with previous reports that mouse prospermatogonia re-enter the cell cycle before P3 (Drumond et al., 2011; Nagano et al., 2000). Cell populations in clusters 1 and 3 gradually increased during development into P15 (Fig. 3B). Cluster 3 cells contained mRNAs encoding the SSC markers LHX1 and GFRA1, while cluster 1 cells contained mRNAs encoding undifferentiated progenitor cell markers NEUROG3 and DDIT4 (Fig. 3B, Fig. S4E). Thus, cluster 3 may represent newly formed SSCs required for steady-state spermatogenesis, and cluster 1 represents undifferentiated progenitor spermatogonia.

Comparing the UMAP plots of control and *Cnot3*-cKO germ cells, we found that *Cnot3* deletion significantly decreased cluster 3 cells (presumed SSCs) and, to a lesser extent, cluster 1 (presumed undifferentiated progenitor spermatogonia) (Fig. 3D,E, Fig. 3D-F). In addition, the SSC marker *Zbtb16* showed decreased expression in cluster 3, and the progenitor markers *Neurog3* and *Ddit4* showed decreased expression in cluster 1 (Fig. 3F). These results are consistent with our conclusions based on immunofluorescence staining (Fig. 2) and provided additional support to the notion that CNOT3 is required for the maintenance of SSCs.

### *Cnot3* is required for SSC maintenance *in vitro*

**Fig. 4.**
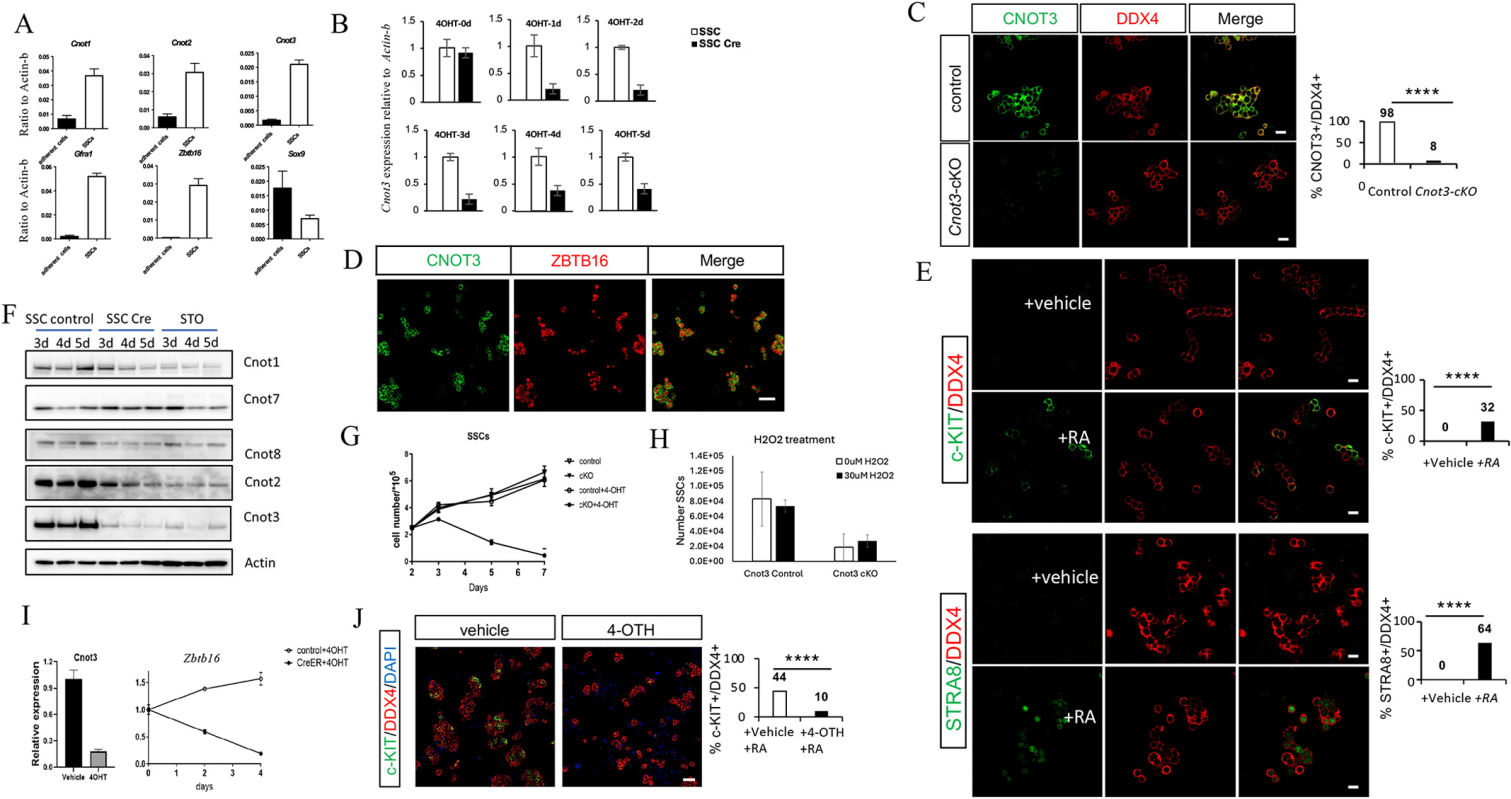
**CNOT3 is required for SSC maintenance and differentiation *in vitro*.** (A) Expression levels determined by real-time RT-PCR of *Cnot1*, *Cnot2*, *Cnot3*, *Gfra1*, *Zbtb16* and *Sox9* genes relative to β-actin in adherent cells and SSCs derived from control testis. Mean±s.e.m. from three independent measurements are shown. (B) Expression level of *Cnot3* relative to β-actin determined by real-time RT-PCR in SSCs derived from control (SSC) and *Cnot3-*cKO (SSC-Cre) and 0-5 days after 4-OHT treatment. Mean±s.e.m. from three independent measurements are shown. (C) Single and merged channels of SSCs derived from control and *Cnot3*-cKO testis showing CNOT3 (green) and DDX4 (red) expression. Cells were treated with 4-OHT for 72 h. The percentage of double-positive cells from at least three independent biological measurements are shown. ****P<0.0001 (two-tailed Fisher's test). (D) Single and merged channels of SSCs derived from control testis showing CNOT3 (green) and ZBTB16 (red) expression. (E) Single and merged channels of SSCs derived from control testis showing KIT (green) and DDX4 (red) expression (top) and STRA8 (green) and DDX4 (red) expression (bottom). Cells were treated with retinoic acid (RA; 1 μM) or vehicle for 24 h. The percentage of double-positive cells from at least three independent biological measurements is shown. ****P<0.0001 (two-tailed Fisher's test). (F) The protein level of CCR4-NOT subunits was detected by immunoblotting in control and *Cnot3*-cKO SSCs. Cells were treated with 4-OHT for 72 h and collected at the indicated time points. d, days after treatment. Feeder cells (STO) were used as a control. (G) The survival curve of control and *Cnot3*-cKO SSCs was determined. Cells were treated with vehicle or 4-OHT for 48 h. Then cells were replated at 2.5×10^5^ cells/well. Cell numbers were determined using a hemocytometer at the indicated time points. Mean±s.e.m. from three independent measurements are shown. (H) Number of SSCs after H_2_O_2_ treatment. Control and *Cnot3*-cKO SSCs were treated with 4-OHT for 48 h. H_2_O_2_ was added for 3 h to the cells, and cells were counted 48 h later. (I) Relative expression level of *Cnot3* and *Zbtb16* genes in control and *Cnot3*-cKO SSCs treated with 4-OHT for 48 h at the indicated time points. Mean±s.e.m. from three independent measurements are shown. (J) Single and merged channels of SSCs derived from *Cnot3*-cKO testis showing KIT (green) and DDX4 (red) expression with DAPI. Cells were treated with 4-OHT or vehicle for 72 h and with RA (1 µM) for 24 h before immunofluorescence staining on day 5. The percentage of double-positive cells from at least three independent biological measurements are shown. ****P<0.0001 (two-tailed Fisher's test). Scale bars: 20 μm in C,E; 50 μm in D,J.

To further test the role of *Cnot3* in SSCs, we derived *in vitro* germ cell cultures enriched for undifferentiated spermatogonia from *Cnot3*^flox/flox^ (control SSCs) and *Cnot3*^flox/flox^; *Ubc*-creER (*Cnot*-cKO SSCs) mice using an established protocol (Brinster and Avarbock, 1994; Brinster and Zimmermann, 1994; Kubota and Brinster, 2008). Real-time RT-PCR documented that the cultured SSCs highly expressed the undifferentiated germ cell markers *Gfra1* and *Zbtb16* but not the Sertoli cell marker *Sox9* (Fig. 4A). In addition, the mRNA level of *Cnot3* was significantly higher in SSCs than the adherent somatic cells from mouse testis (Fig. 4A). Real-time RT-PCR showed efficient *Cnot3* deletion upon 4-OHT treatment (Fig. 4B). The SSC identity of the cultured cells was further confirmed by immunofluorescence staining of the pan germ cell marker DDX4 and the undifferentiated spermatogonia marker ZBTB16 (Fig. 4C,D). In response to RA treatment, cultured undifferentiated spermatogonia were also able to transit differentiating spermatogonia, which express both KIT and STRA8 (Fig. 4E). After 4-OHT treatment, CNOT3 protein was lost by 72 h in *Cnot3*-cKO spermatogonia (Fig. 4F). The deletion of *Cnot3* decreased CNOT1 and CNOT2 protein levels without affecting the enzymatic module CNOT7, or CNOT8 (Fig. 4F). We found that *Cnot3* deletion impaired the proliferation and/or viability of cultured undifferentiated spermatogonia (Fig. 4G). More importantly, the deletion resulted in the downregulation of the key SSC marker gene *Zbtb16*, indicating that CNOT3 is required to maintain undifferentiated spermatogonia, including SSCs (Fig. 4I). Finally, we treated the cultured SSCs with RA to induce differentiation. In comparison to WT SSCs, *Cnot3*-deleted SSCs could not activate KIT, encoded by a known RA-response gene expressed in differentiating spermatogonia (Fig. 4J). This result suggested that *Cnot3* deletion compromises SSC differentiation. Therefore, similar to our *in vivo* findings, these results indicate that CNOT3 is required for the maintenance and function of undifferentiated spermatogonia and SSCs *in vitro*.

### *Cnot3* deletion in SSCs affects its transcriptome profile

**Fig. 5.**
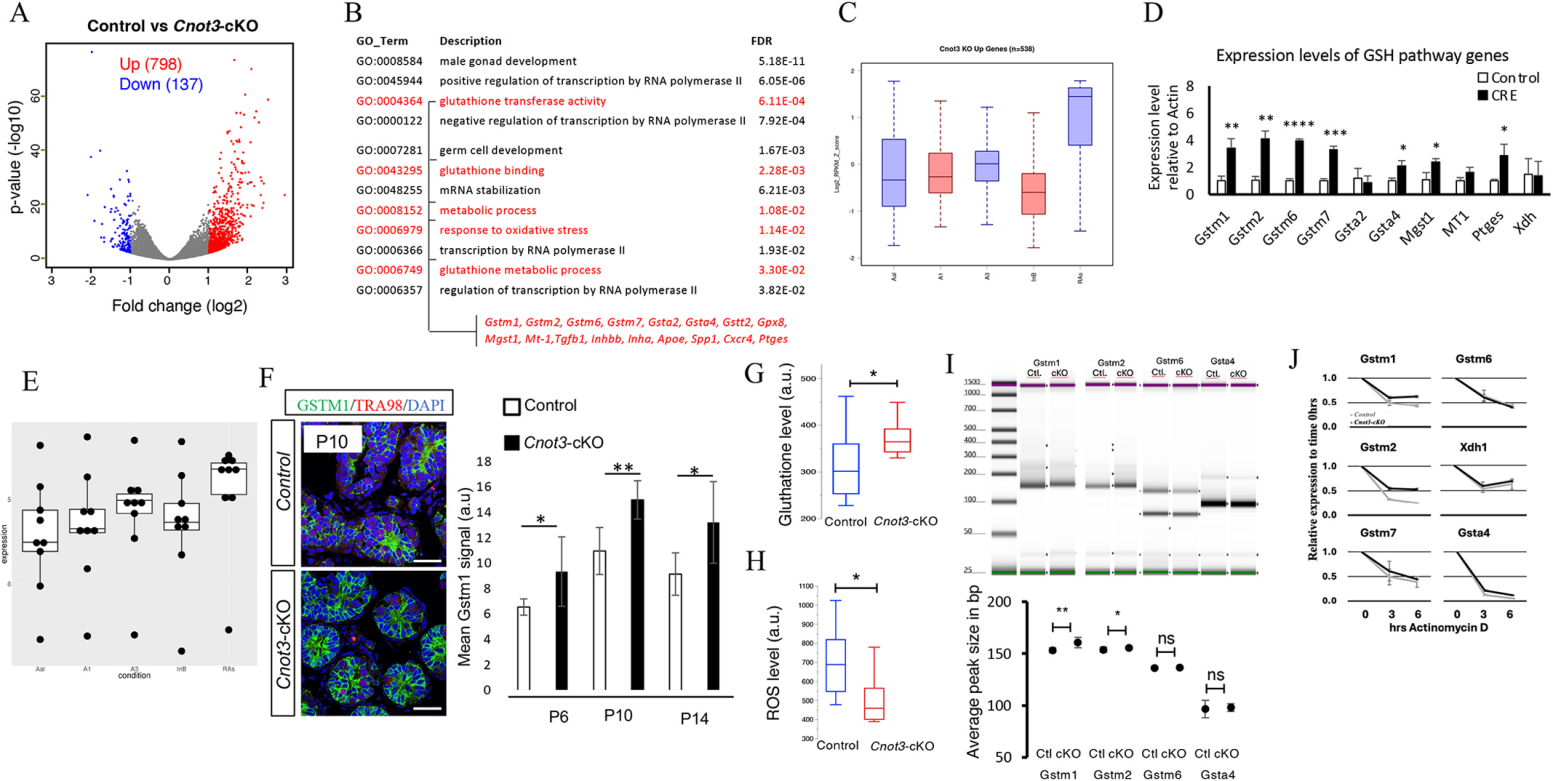
***Cnot3* deletion affects spermatogenic gene expression and GSH/ROS balance.** (A) Volcano plot (log2 fold change versus −log10 *P*-value) of RNA-seq data from control and *Cnot3*-cKO ID4-EGFP^+^ undifferentiated spermatogonia at P8 (three biological replicates), identifying 798 and 137 genes upregulated and downregulated, respectively. (B) Gene ontology analysis of upregulated genes in *Cnot3*-cKO ID4-EGFP^+^ undifferentiated spermatogonia at P8. Pathways and genes involved in the GSH/ROS pathway are indicated in red. (C) Box plot showing the expression profile of the genes upregulated in *Cnot3*-cKO ID4-EGFP^+^ SSCs in undifferentiated spermatogonia stages A aligned (Aal) and A1 and differentiating spermatogonia A3, Intermediate B (InB) and preleptotene stages (RAs) (Kirsanov et al., 2023). (D) Expression level of GSH pathway genes relative to expression of *Actb* in ID4-EGFP^+^ spermatogonia from control and *Cnot3*-cKO. Mean±s.e.m. from three independent measurements is shown *P<0.05, **P<0.01, ***P<0.001, ****P<0.0001 (two-tailed, unpaired Student's *t*-test). (E) Box plot showing the expression profile of the GSH pathway genes *Gstm1*, *Gstm2*, *Gstm6*, *Gstm7*, *Gclc*, *Gsta4*, *Mgst1*, *Mt1* and *Ptges* in undifferentiated spermatogonia stages A aligned (Aal) and A1 and differentiating spermatogonia A3, Intermediate B (InB) and preleptotene stages (RAs) (Kirsanov et al., 2023). (F) Merged channels of single confocal sections of P10 control and *Cnot3*-cKO testis showing GSTM1 (green) and TRA98 (red) expression with DAPI (blue). Scale bar: 50 μm. Average±s.e.m. of mean GSTM1 signal intensity of positively labeled SSCs from at least three independent biological measurements are shown *P<0.05, **P<0.01 (two-tailed, unpaired Student's *t*-test). (G) Box and whisker plot of GSH levels measured by flow cytometry in control and *Cnot3*-cKO ID4-EGFP^+^ undifferentiated spermatogonia at P8. *P<0.05 (two-tailed, unpaired Student's *t*-test). (H) Box and whisker plot of ROS levels measured by flow cytometry in control and *Cnot3*-cKO ID4-EGFP^+^ undifferentiated spermatogonia at P8. *P<0.05 (two-tailed, unpaired Student's *t*-test). (I) TapeStation electrophoresis of poly(A) tail generated by the extension poly(A) test (ePAT), for the indicated transcripts at P8. *P<0.05, **P<0.01 (two-tailed, unpaired Student's t-test). ns, not significant (*P*>0.05). (J) Examination of mRNA stability for the indicated genes. *Cnot3*-cKO SSCs were treated with (KO) or without 4-OHT (WT) for 48 h. Actinomycin D was added to the cells, and mRNA level was measured by RT-qPCR at the indicated time points. a.u., arbitrary units.

To understand how *Cnot3* deletion leads to dramatic loss of undifferentiated spermatogonia and SSCs, we examined transcriptome changes between control and *Cnot3*-cKO spermatogonia. We first compared the WT and *Cnot3* KO Cluster 3 SSCs. However, due to the limited sensitivity of scRNA-seq, both the number and fold change of differentially expressed genes (DEGs) were rather modest. Therefore, we enriched the SSCs using known SSC markers. Since ID4 is an established marker of SSCs in the developing testis (Chan et al., 2014; Cheng et al., 2020; Hermann et al., 2018; Oatley et al., 2011; Velte et al., 2019), we generated *Cnot3*^flox/flox^; *Ddx4*-creER; *Id4-eGfp* mice (Helsel et al., 2017; Zheng et al., 2016). We isolated single-cell suspensions from P8 testes (since *Cnot3* was effectively deleted by this point and CNOT3 protein was undetectable), and collected ID4-EGFP^+^ spermatogonia by flow cytometry for bulk RNA-seq. Using fold change >2 and adjusted *P*<0.01 in the RNA-seq analysis, we identified 798 transcripts that were upregulated and 137 that were downregulated in *Cnot3*-cKO testes compared to controls (Fig. 5A, Table S1). Such a distinct imbalance between the up- and downregulated mRNA levels is consistent with the known function of *Cnot3* in promoting mRNA degradation (Morita et al., 2011), and suggests that CNOT3 normally represses gene expression in SSCs. Based on gene ontology analysis, we found that these upregulated genes were significantly enriched for terms related to RNA metabolic processes and germ cell development (Fig. 5B).

Next, we collated transcripts that were increased in *Cnot3*-cKO ID4-EGFP^+^ spermatogonia and examined their levels during spermatogonial differentiation. This was done using our recently published RNA-seq dataset from germ cells isolated from mice with synchronized steady-state spermatogenesis: undifferentiated spermatogonia and from spermatogonia at the onset (type A1), midpoint (type A3) and end (type intermediate/B) of differentiation, and preleptotene spermatocytes entering meiosis (Kirsanov et al., 2023) (Fig. 5C). We found that mRNAs that increased in *Cnot3*-cKO ID4-EGFP^+^ cells tended to be upregulated between undifferentiated and early/mid-stages (type A1-A3) of spermatogonial differentiation. This observation suggested these *Cnot3*-repressed transcripts are normally upregulated during SSC differentiation. Thus, it is conceivable that CNOT3 normally promotes the degradation of transcripts involved in spermatogonial differentiation to maintain SSC identity and function.

### *Cnot3* deletion upregulates the GSH redox pathway

Interestingly, among the CNOT3-repressed transcripts in our RNA-seq analyses, we noted that transcripts encoding proteins in the GSH redox pathway were significantly over-represented (Fig. 5B,D). The GSH pathway protects cells from oxidative damage by reactive oxygen species (ROS) to promote cell proliferation and differentiation (Meister, 1988; Sies, 1991). The glutathione S-transferase alpha 4 and m1 (*Gsta4* and *Gstm1*) genes were overexpressed in SSCs compared to differentiating spermatogonia, suggesting a role for the oxidative stress response pathway in regulating the survival and proliferation of SSCs (Kokkinaki et al., 2009; Suzuki et al., 2021). A balance in ROS levels mediated by GSH is crucial for spermatogonial maintenance and differentiation – basal ROS levels are essential for self-renewal and proliferation of SSCs (Morimoto et al., 2013, 2019), while significantly elevated ROS levels are correlated with male infertility due to their damaging impacts on spermatogonial maintenance and differentiation (Agarwal and Sekhon, 2010; Ishii et al., 2005; Lenzi et al., 1993; Nakamura et al., 2010; Takubo et al., 2006). Thus, we hypothesized that *Cnot3* deletion would disrupt the balance between GSH function and ROS levels and thereby impair SSC maintenance. To test the hypothesis, we first examined transcript levels of several genes of the GSH pathway (*Gstm1*, *Gstm2*, *Gstm6*, *Gstm7*, *Gclc*, *Gsta4*, *Mgst1*, *Mt1*, *Ptges*) in ID4-EGFP^+^ cells isolated from control and *Cnot3*-cKO testes. We found that *Cnot3* deletion led to the upregulation of seven out of the ten transcripts. Moreover, based on their expression in undifferentiated spermatogonia, A1, A3 and In/B differentiating spermatogonia, and preleptotene spermatocytes from our previous study (Kirsanov et al., 2023), these GSH pathway-encoding transcripts were mostly upregulated during spermatogonial differentiation (Fig. 5E). In addition, we examined the protein level of GSTM1 in WT and *Cnot3*-cKO testis at P6, P10 and P14, and found an increase of the GSTM1 signal in *Cnot3*-cKO SSCs (Fig. 5F). These observations suggest that CNOT3 represses the GSH pathway in SSCs, and GSH pathway activation may be a necessary step for spermatogonial differentiation. Finally, we examined GSH and ROS levels with flow cytometry using live cell fluorescent dyes in cell suspensions freshly prepared from control and *Cnot3*-cKO P8 testes. In ID4-EGFP-high cells, GSH levels were significantly increased while ROS levels were significantly decreased in the *Cnot3*-cKO samples (Fig. 5G,H, Fig. S5), consistent with the role of GSH in preventing ROS accumulation.

Because CNOT3 is part of the CCR4-NOT mRNA deadenylase complex, we tested whether it may regulate the poly(A) tail length of the GSH pathway gene transcripts using the extension Poly(A) Test (ePAT) (Janicke et al., 2012). We observed that *Cnot3* deletion led to an increase in poly(A) tail length of *Gstm1* and *Gstm2* mRNAs, but no change for *Gstm6* and *Gsta4* transcripts (Fig. 5I), suggesting that only a subset of mRNAs was affected. To investigate further the consequence of this, we examined the stability of GSH pathway mRNAs in cultured SSCs (Fig. 5J) by RT-qPCR after transcription inhibition. Upon *Cnot3* deletion, we observed an increase in the half-life of *Gstm1*, *Gstm2* and *Gstm7* transcripts, but no changes for *Gstm6, Xdh* and *Gsta4* transcripts. Thus, *Cnot3* deletion appears to promote poly(A)-tail lengthening and stabilization of *Gstm1* and *Gstm2* mRNAs, suggesting a role for *Cnot3* in promoting the deadenylation and degradation of a subset of GSH pathway transcripts.

To test the functional significance of the observed changes in ROS levels, we treated cultured SSCs with hydrogen peroxide to elevate ROS and tested whether the treatment could mitigate the cell loss observed upon *Cnot3* deletion (Barati et al., 2015; Mori et al., 2021). We found that 30 µM H_2_O_2_ treatment did not affect the control SSCs (Barati et al., 2015; Mori et al., 2021), but led to a slight increase in cell number in *Cnot3* deletion SSCs (Fig. 4H). However, this increase was not statistically significant under our experimental conditions. While our H₂O₂ rescue experiment did not reverse SSC loss *in vitro*, it suggests that ROS alone are not the sole cause of SSC depletion. Previous studies highlight that basal ROS levels are crucial for SSC self-renewal and proliferation, and, *in vivo*, ROS depletion reduces SSC numbers (Ishii et al., 2005; Nakamura et al., 2010; Takubo et al., 2008; Morimoto et al., 2013). However, our results indicate that the *in vitro* environment may not fully capture the complexity of ROS signaling *in vivo*. It is possible that other factors that are misregulated after Cnot3 deletion, such as signaling molecules and the testicular microenvironment, may make SSCs more responsive to ROS *in vivo*. Thus, while ROS are important for SSC function, their role likely depends on the specific cellular and environmental context.

In summary, our findings revealed that in the absence of CNOT3 upregulation of transcripts encoding GSH pathway components leads to an accumulation of GSH and a reduction of ROS. Therefore, it is conceivable that the reduced ROS level in *Cnot3* deletion impairs spermatogonial maintenance, contributing partly to the phenotype of germ cell loss and infertility *in vivo*. It is worth noting that GSH pathway genes only account for a small fraction of all upregulated genes upon *Cnot3* deletion. Many of the other upregulated factors may also be involved in the SSC maintenance defects.

## DISCUSSION

The developmental program of spermatogenesis, which is essential for lifelong male fertility, relies on the continual function of SSCs, which balance self-renewal divisions with those that produce undifferentiated progenitors that eventually become committed to generating sperm (de Rooij and Russell, 2000; Oatley and Brinster, 2008). The formation and maintenance of these undifferentiated spermatogonial populations depend on a gene expression program regulated by a coordinated balance between both transcriptional and post-transcriptional mechanisms. The germ cell type-specific transcriptome is shaped by the coordinated processes of transcription and RNA decay. Here, we discovered that, in mice, CNOT3, a subunit of the CCR4-NOT mRNA deadenylase complex, plays a crucial role in the regulation of SSCs and their potential for self-renewal. CNOT3 is particularly abundant in undifferentiated spermatogonia, and its deletion in spermatogonia led to germ cell loss and infertility. In the absence of CNOT3, transcripts associated with factors involved in SSC differentiation are de-repressed. These findings suggest that CNOT3, likely through the CCR4-NOT complex, facilitates the degradation of differentiation gene transcripts to maintain the spermatogonial pool and ensure the progression of spermatogenesis. This highlights the importance of CNOT3 during male germ cell development. Furthermore, the present study on the postnatal male germline, taken together with our previous work in embryonic stem cells (Hu et al., 2009; Zheng et al., 2012), suggests that CNOT3 and CCR4-NOT-mediated post-transcriptional regulation can protect the identity and self-renewal of stem cells in different contexts.

Our transcriptome analyses showed that transcripts encoding factors targeted by CNOT3 in SSCs included those in the GSH redox pathway. Elevated ROS levels can cause oxidative stress and negatively affect male fertility by impacting SSC survival and function (Ishii et al., 2005; Nakamura et al., 2010; Takubo et al., 2006). However, basal levels of ROS are also required for SSC self-renewal (Morimoto et al., 2013). Moreover, suppression of ROS levels showed reduced SSC proliferation, while hydrogen peroxide treatment increased SSC self-renewal (Morimoto et al., 2013, 2019). Here, we observed that in SSCs CNOT3 prevents the elevation of GSH levels and the decrease of ROS levels, thus contributing to the integrity of SSC function.

Poly(A) tails at mRNA 3′ ends play an essential role in maintaining mRNA steady-state levels in eukaryotes (Brawerman, 1981; Wickens et al., 1997). Deadenylation, which is the process of removing the poly(A) tail and the initial step in all forms of mRNA decay, is essential for maintaining transcriptome balance (Goldstrohm and Wickens, 2008). The major mRNA deadenylase in eukaryotes is the CCR4-NOT multi-subunit complex, which includes the scaffold protein CNOT1, the NOT-domain subunits CNOT2 and CNOT3, the catalytic subunits CNOT6, CNOT6L, CNOT7 and CNOT8, and other regulatory subunits (Collart and Panasenko, 2012, 2017). Using genetic approaches, previous studies have uncovered important roles for CCR4-NOT-mediated mRNA decay during both embryonic and germ cell development. In embryonic development, CNOT3 is required for the maintenance of pluripotent stem cells (Zheng et al., 2012, 2016). CNOT4 is required for post-implantation development and male meiosis (Dai et al., 2021). *Cnot6/6l* whole-body deletion results in female infertility due to defective maternal mRNA degradation during oocyte maturation. (Berthet et al., 2004; Sha et al., 2018). *Cnot7* deletion impairs spermatogenesis and male fertility, but oogenesis progresses normally (Berthet et al., 2004). Thus, the CCR4-NOT complex can play different roles in different cell types and cellular contexts. While our current study focused on the role of CNOT3 in SSCs, it is conceivable that CNOT3 plays important roles in additional stages of spermatogenesis. Consistent with this notion, *Cnot3* deletion also led to a small decrease in the undifferentiated progenitor cell population (Fig. 3D-F).

There are several examples of mRNA-binding proteins that interact with the CCR4-NOT deadenylase complex in the germline to promote the degradation of mRNAs involved in spermatogonial differentiation. This activity is proposed to prevent premature differentiation and ensure that they remain self-renewing. NANOS2, an RNA-binding protein that promotes RNA degradation, is only expressed in SSCs in the postnatal testis, and mice deficient in NANOS2 have a progressive loss of SSCs, leading to germline extinction (Codino et al., 2021; Sada et al., 2009; Suzuki et al., 2010). Separately, *Dnd1* is expressed from the origin of the germline in primordial germ cells through to postnatal spermatogonia in mice. DND1 directly recruits the CCR4-NOT complex to destabilize mRNAs encoding factors involved in the positive regulation of apoptosis, pluripotency and inflammation. DND1 function is required for the survival of primordial germ cells and SSCs and, in some genetic backgrounds, to prevent the development of testicular germ cell tumors (Cook et al., 2009, 2011; Sakurai et al., 1995; Suzuki et al., 2016; Yamaji et al., 2017). To test the involvement of NANOS2 or DND1, we carried out motif enrichment analysis in the differentially regulated genes after *Cnot3* deletion. However, we did not find any significant enrichment for known NANOS2- and DND1-binding motifs in the 3′-UTR of our gene set. In addition, we also carried out *de novo* motif analysis but failed to identify any significantly enriched motif. As CCR4-NOT can be regulated by its interaction with various factors, including mRNA-binding proteins, BTG/Tob family factors and miRNA silencing pathways, in the future it will be important investigate further how it targets specific mRNA subsets for degradation.

## MATERIALS AND METHODS

### Animals and 4-OHT treatment

*Cnot3*^flox/flox^ and *Ddx4*-CreER transgenic mice were bred to generate *Cnot3*^flox/flox^; *Ddx4*-creER mice. *Cnot3*^flox/flox^*; Ddx4*-creER; *Id4-eGfp* mice were derived by breeding *Cnot3*^flox/flox^; *Ddx4*-creER and *Id4-eGfp* mice (Zheng et al., 2016). The genotypes of offspring were determined by qRT-PCR by Transnetyx, Inc. (Zheng et al., 2016). For adult mice, 4-week-old *Cnot3*^flox/flox^; *Ddx4*-creER mice were dosed with 200 mg/kg 4-OHT for three consecutive days. Testes were collected from both 6- and 8-week-old mice. Neonatal mice were given daily subcutaneous injections (25 ga needles) of 25 µg of 4-OHT from P1 to P3. Testes were collected at P6, P8, P10, P14 and P21. All animal procedures were approved by the National Institutes of Health Animals Care and Use Committee and were performed in accordance with an approved National Institute of Environmental Health Sciences animal study proposal.

### Breeding study

Adult male mice (8 weeks of age) were housed individually, and two 6-week-old female mice (C57B1/6) were placed with each male for 1 month. After each of the 1-month mating periods, females were held for an additional 3 weeks to quantify the number of litters and offspring produced.

### *In vitro* SSC culture

SSCs were derived from *Cnot3*^flox/flox^ and *Cnot3*^flox/flox^; *Ddx4-creER* mice, which were bred to a DBA/2 background for more than four generations following a published protocol (Kubota and Brinster, 2008). Briefly, testes were dissected from mice at the age of P5-P8. After the tunica was removed, seminiferous tubules were digested with 7 mg/ml DNase I solution and 0.25% Trypsin-EDTA into a single-cell suspension. Then, SSCs were enriched by 30% Percoll fractionation and then plated on mitomycin C-treated STO cells (SNL76/7 cells) (McMahon and Bradley, 1990). SSCs were maintained in MEMα-based Mouse Serum-Free Medium (Kubota and Brinster, 2008). To induce *Cnot3* deletion, we added 4-OHT freshly prepared in ethanol as per the manufacturer's instructions (Stem Cell Technologies, 72662) to the medium at 0.1 µM at the indicated time points.

To generate a survival curve of control and *Cnot3*-cKO SSCs, cells were treated with vehicle or 4-OHT for 48 h. Then, cells were replated at 2.5×10^5^ cells/ well. Cell numbers were determined using a hemocytometer at the indicated time points.

To induce SSCs to enter meiosis, cells were treated with 4-OHT or vehicle for 72 h and with RA (1 μM; R2625, Sigma-Aldrich) freshly prepared in DMSO for 24 h before immunofluorescence staining on day 5.

The H_2_O_2_ treatment was performed following a previous published report (Barati et al., 2015; Mori et al., 2021). Two days after 4-OHT treatment for *Cnot3* conditional deletion, *Cnot3* control and cKO SSCs were treated with 30 µM H_2_O_2_ or vehicle for 3 h.

To measure mRNA half-life in a subset of genes after *Cnot3* deletion, *Cnot3* cKO SSCs were treated with 4-OHT (KO) or vehicle (control). Actinomycin D was added 48 h after treatment with (*Cnot3*-cKO) or without 4-OHT (*Cnot3*-Control), and cells were collected at 0, 3 and 6 h after actinomycin D addition for RT-qPCR analysis.

### Extension Poly(A) test (ePAT)

Briefly, 1 μg of total RNA, 1 μl of RNA 3′ Adapter and H_2_O were combined in an 8 μl volume, heat denatured at 70°C for 2 min and cooled on ice for at least 1 min. We added 1 μl of 10x RNA Ligase, RNase inhibitor and RNA ligase 2 KQmut (New England Biolabs, M0373S) and incubated at 28°C for 1 h with shaking at 1000 rpm. We mixed in 4 μl of Maxima RT buffer, 1 μl dNTP at 10 mM, 2 μl 9T oligo at 10 μM, 2 μl H_2_O and 1 μl Maxima RT (Thermo Fisher Scientific, EP0741) and ran a reverse transcription reaction for 30 min at 50°C followed by 5 min at 85°C and hold at 12°C. We then diluted the cDNA at 1:5 prior to perform PCR reactions using forward gene-specific primers and reverse 12T oligo for PolyA-PCR reaction and forward and reverse gene-specific primers for control PCR reactions, with a Tm of 55°C, an extension of 1 min and for 30 to 35 cycles. The PCR products were then run on a TapeStation system (Agilent) to evaluate the poly(A) tail length. The average peak size of the ePAT PCR products were measured in three independent control and cKO samples using a TapeStation (Agilent). Differences between samples were assessed by two-tailed, unpaired Student's *t*-test (QuickCalcs; https://www.graphpad.com/quickcalcs).

Primers used for ePAT were: Gstm1 pA PCR-F1, cttagtgctagccctccctagag; Gstm2 pA PCR-F1, ggcctggcctgagagattagatctg; Gstm6 pA PCR-F1, ctgcactttgagcagcttagccc; Gsta2 pA PCR-F1, gctgcattgatggagccacag.

### Tissue processing

Tissues were fixed with 4% paraformaldehyde in 1× PBS overnight at 4°C. For frozen sections, tissues were dehydrated with 30% sucrose and incubated at 4°C until the tissue sank, embedded in O.C.T. and cryosectioned at 10 μm onto glass microscope slides, which were then stored at −80°C until use. For paraffin sections, tissues were dehydrated through an ethanol gradient and embedded in paraffin wax using standard methods. Paraffin-embedded specimens were sectioned at 5 μm onto glass microscope slides, which were then stored at room temperature until use.

### Histology

Paraffin sections of 5 μm thickness were stained with Hematoxylin and Eosin using standard methods by the Immunohistochemistry Support-Pathology Support Group at NIEHS. Hematoxylin and Eosin-stained slides were scanned using Leica Biosystems digital slide scanner by the Imaging Sciences and Artificial Intelligence Group at NIEHS. Representative images were captured using ImageScope through eSlide Manager (Leica Biosystems).

### Immunohistochemistry

Paraffin sections were incubated with antigen unmasking solution (H-3300, Vector Laboratories) for antigen retrieval and 3% H_2_O_2_ (H325, Fisher Scientific) to inactivate endogenous peroxidase. Sections were incubated with the blocking reagent (goat serum) and followed with homemade rabbit anti-mouse CNOT3 (1:1500; Zheng et al., 2012; Table S2) at room temperature for 1 h. Sections were washed three times and then incubated with biotinylated goat anti-rabbit IgG (H+L) (see Table S2) or 30 min at room temperature. Then, ABComplex/HRP (VECTASTAIN ABC-HRP Kit, Vector Laboratories) and DAB substrate (SK4100, Vector Laboratories) were applied to the sections at room temperature. The sections were counterstained with Hematoxylin (HHS16, Sigma-Aldrich), dehydrated, and mounted for imaging. Slides were scanned using a Leica Biosystems digital slide scanner by the Imaging Sciences and Artificial Intelligence Group at NIEHS. Representative images were captured using ImageScope through eSlide Manager (Leica Biosystems).

### Immunofluorescence staining

Cryosections were hydrated with 1× PBS and then permeabilized in blocking buffer (3% bovine serum albumin, 0.1% Triton X-100 in 1× PBS) for 30 min at room temperature. Antibodies (see Table S2) were applied at appropriate dilutions in the blocking buffer and incubated overnight at 4°C. The slides were washed the next day (0.05% Triton X-100 in 1× PBS) for 5 min, followed by two 10 min washes in 1× PBS. Primary antibodies were omitted in negative controls. The primary antibodies were detected by incubating slides for 2 h at room temperature with the corresponding secondary antibodies (see Table S2). After incubation, the slides were washed (0.05% Triton X-100 in 1× PBS) for 5 min, followed by two 10 min washes in 1× PBS, and counterstained with DAPI. VECTASHIELD (H-1000-10, Vector Laboratories) was used as antifading mounting solution. For paraffin sections, sections were deparaffinized and rehydrated prior to antigen retrieval and standard immunostaining procedures. To perform immunofluorescence staining on SSCs, SSCs were seeded on STO feeders, which were prepared 1 day before on Millicell EZ Slides (PEZGS0816, Millipore). SSCs were treated with 4-OHT at 0.1 µM or RA at 1 µM or vehicle for the indicated time. Cells were fixed with 4% paraformaldehyde in PBS for 10 min at room temperature prior to standard immunostaining procedures. Confocal images were taken with a Zeiss LSM 710 inverted microscope (Carl Zeiss Inc.) using the 405 nm, 488 nm and 594 nm laser lines for excitation. A Plan-APOCHROMAT 63×/1.4 Oil DIC or a Plan-APOCHROMAT 20×/0.8 objective was used for image collection. The quantitation of positively labeled cells were blindsided and manually performed using ZEISS ZEN software (version 2.3, SP1) for visualization. The level of GSTM1 mean intensity signal was measured using Fiji version 2.14.0/1.53 s. Differences between samples were assessed with the Smirnov–Kolmogorov test, two-tailed, unpaired Student's *t*-test and two-tailed Fisher's test (QuickCalcs; http://www.graphpad.com/quickcalcs/index.cfm).

### Immunoblotting

*In vitro* cultured SSCs were lysed in a NuPAGE LDS Sample Buffer (Invitrogen, NP0007) at the same cell concentration. An equal amount of proportion was loaded in each lane (Zheng et al., 2012). Antibodies are listed in Table S2.

### Isolation of ID4-EGFP cells by fluorescence-activated cell sorting

Testes were dissected from 4-OHT-treated neonatal mice (*Cnot3*^flox/flox^;*Id4-eGfp*, and *Cnot3*^flox/flox^; *Ddx4*-creER, ID4-EGFP). Tunica were removed and released seminiferous tubules were placed in a dish with 4.5 ml 0.25% trypsin (25200056, Thermo Fisher Scientific) and 0.5 ml Accutase (A1110501, Thermo Fisher Scientific). Tubules were dissociated by pipetting up and down several times and incubated in a 37°C CO_2_ incubator for 5 min. Another 1 ml Accutase was added, and pipetting and incubation steps were repeated twice until the clumps were dispersed into a single-cell suspension. The single-cell suspension was placed on ice, and ID4-EGFP^+^ cells were collected by a BD FACSAria II Cell Sorter (BD Biosciences). ID4-EGFP^+^ cell pellets were centrifuged at 300 ***g*** for 20 min and stored in RNA lysis buffer (7326820, Bio-Rad; Aurum™ Total RNA Mini Kit) at −80°C until use.

### The cellular level of ROS and GSH measurement

Single-cell suspensions were prepared from P8 ID4-EGFP control and ID4-EGFP-*Cnot3*-cKO testes. The cells were stained using a CellROX™ Deep Red Flow Cytometry Assay Kit (C10491, Thermo Fisher Scientific) for ROS level and stained with ThiolTracker™ Violet (GSH detection reagent) (T10095, Thermo Fisher Scientific) to detect reduced GSH according to the manufacturer's protocol. The cells were analyzed on a BD LSRFortessa™ Cell Analyzer (BD Biosciences). All measurements were independently performed and at least in triplicate. Differences between samples were assessed with two-tailed, unpaired Student's *t*-test (QuickCalcs; https://www.graphpad.com/quickcalcs).

### Real time RT-PCR

Total RNA was isolated with TRIZOL reagent (Invitrogen) and treated with TURBO DNA-free (Ambion) as per manufacturer's recommendations. cDNA synthesis was produced with oligo dT primers and SuperScript III Reverse Transcriptase (Invitrogen). The cDNAs were diluted in water for downstream PCR reactions using appropriate primers (Table S3) and SsoFast EvaGreen Supermix (Bio-Rad) and performed in triplicate. Real-time PCR Bio-Rad CFx96 Real-Time PCR System was used to detect Eva Green. Standard curves were generated using a serial dilution, and *Actb* was used for normalization. Relative quantitation was analyzed using ΔΔCt methods. All measurements were independently performed and at least in triplicate. Differences between samples was assessed with two-tailed, unpaired Student's *t*-test (QuickCalcs; https://www.graphpad.com/quickcalcs).

### RNA-seq data analysis

ID4-EGFP^+^ spermatogonia were collected by flow cytometry from P8 testes of control and *Cnot3*-cKO mice. Total RNA was isolated using the Bio-Rad Aurum Total RNA Mini Kit as per the manufacturer's recommendations. RNA-seq library was constructed using the Illumina TruSeq Stranded Total RNA kit and sequenced on a NextSeq 500 system (Illumina).

Reads were quality-filtered, only keeping those with a mean Phred quality score of 20 or greater. Reads were aligned to the mm10 assembly using STAR version 2.6.0c (Dobin et al., 2013). Gene read counts were obtained using the GENCODE annotation version M24 and the featureCounts tool from the Subread package version 1.5.1 (Liao et al., 2014). DEGs were identified using DESeq2 version 1.14.1 (Love et al., 2014). Gene ontology enrichment analysis was performed using goSTAG (Bennett and Bushel, 2017) version 1.12.1.

### scRNA-seq data analysis

CD9^+^/THY1^+^ testis cells were collected by flow cytometry from P8 testes of control and *Cnot3*-cKO mice. scRNA-seq libraries were prepared with the 10x Genomics Chromium Single Cell 3′ Kit v3.1 and sequenced on NextSeq 500 and NovaSeq 6000 systems (Illumina).

#### scRNA-seq data processing

Raw read processing was carried out using the Cell Ranger Single-Cell Software Suite (version 3.0.1, 10x Genomics Inc.). Briefly, the demultiplexed FASTQ files (paired-end; Read 1: 150 bp; Read 2: 150 bp) were generated using the CellRanger mkfastq command. The primary data analyses, which included alignment, filtering, barcode counting and unique molecular identifier (UMI) quantification for determining gene transcript counts per cell (generated a gene-barcode matrix), quality control, clustering and statistical analysis, were performed using CellRanger count command. Genes were annotated using Ensembl build 93.

#### Single-cell gene expression quantification and filtering

Raw gene expression matrices generated per sample using CellRanger were imported into R (version 4.0.0) and converted to a Seurat object using the Seurat R package (version 3.4) (Satija et al., 2015). Dead cells and doublets were removed. The first, the total number of UMIs and genes, and the percentage of UMIs derived from the mitochondrial genome for each cell were counted. Then, cells that had over 10% UMIs derived from the mitochondrial genome were discarded. Next, the upper bound was calculated as mean plus two s.d. and the lower bound as mean minus two s.d. for both the total UMIs and genes, respectively. Finally, cells with total UMIs or genes outside of the upper and lower bounds were removed.

#### Data integration (CCA) and determination of the major cell types

The remaining cells were integrated together, and batch effects were correct using CCA of Seurat (Satija et al., 2015). The main cell types were identified on the basis of predicted and known marker genes acquired from the SingleR (https://github.com/LTLA/SingleR) %20and the CellMarker database (http://biocc.hrbmu.edu.cn/CellMarker/).

#### Data integration of germ cells and determination of the major cell types

All germ cell raw expression profiles were extracted and integrated together and batch effects were corrected using RunFastMNN from the R package ‘batchelor’ (Haghverdi et al., 2018). First, for each sample, gene expression matrices were normalized to total cellular read count and Cell-Cycle scores were calculated using Seurat CellCycleScoring function. Then, the Seurat SCTransform function was applied for the normalized data to remove the cell cycle effect and select 2500 highly variably genes (HVGs) for downstream analysis. HVGs were checked and mitochondrial genes removed. Following that, principal component analysis was scaled and re-calculated for the cleaned HVGs. The RunUMAP function was then applied for UMAP dimensional reduction. The FindNeighbors function was used to construct a shared nearest neighbor graph, and the FindClusters function with ‘resolution=0.5’ parameter was carried out to cluster cells into different groups. The main cell types were identified on the basis of predicted and known marker genes. For SSC data analysis, all SSC raw expression profiles were extracted and integrated together and batch effects were correct using RunFastMNN. The following analysis was the same as for all germ cells.

### Identification of marker genes and DEGs

To identify marker genes for these cell types, we compared the gene expression values of cells from the cluster of interest to that of cells from the rest of the clusters using the Seurat FindMarkers function with default parameters of the ‘MAST’ test (Finak et al., 2015). Marker genes were defined based on the following criteria: (1) the average expression value in the cluster of interest was at least 1.2-fold higher than the average expression in the rest of clusters; (2) there were greater than 10% of cells in the cluster of interest that were detectable; and (3) marker genes had the highest mean expression in the cluster of interest compared to the rest of clusters.

To identify DEGs between two group of cells, e.g. N-acute myeloid leukemia versus healthy donors, the Seurat FindMarkers function with method ‘MAST’ were applied for two group of cells with parameters ‘min.pct=0.01, logfc.threshold=0.01’.

### Gene set enrichment analysis

For marker genes and DEG lists, gene ontology and pathway analyses were performed by the R package ClusterProfile (version 3.18.1) (Wu et al., 2021). Terms with false discovery rate <0.05 were regarded as significant enrichment.

## Supplementary Material

10.1242/develop.204557_sup1 Supplementary information

Table S1. List of differentially expressed genes (DEGs) with a fold change >2 and adjusted P<0.01, when comparing RNA-seq data set from FACS sorted ID4-EGFP+ spermatogonia *Cnot3*-Control versus *Cnot3*cKO. 798 transcripts were upregulated (DEGs_UP) and 137 were downregulated (DEGs-DOWN) in *Cnot3*-cKO ID4-EGFP+ spermatogonia as compared to controls.
