# Supplementary material for: Cnot3 is required for male germ cell development and spermatogonial stem cell maintenance
Source: Development. 2025 Aug 15;152(15):dev204557. doi: 10.1242/dev.204557 (PMC12401510; doi:10.1242/dev.204557)
Supplement: Supplementary information [file develop-152-204557-s1.pdf]

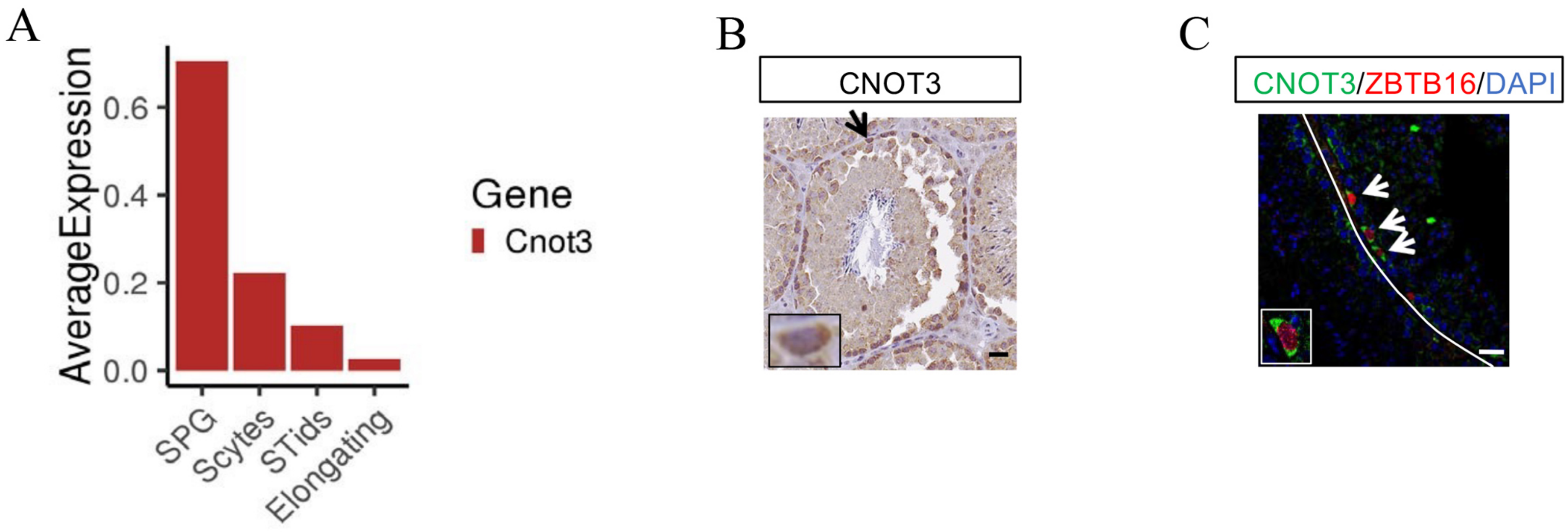

Fig. S1. *Cnot3* deletion results in germ cell loss and male infertility

(A) Analyses of the abundance of *Cnot3* transcripts in Spermatogonia (SPG), Spermatocytes (Scytes), Spermatids (STids) and

(B) Sections of testis of 8wo WT male showing CNOT3 (brown) and hematoxylin.

(C) Merged channels of single confocal sections of 8wo testis showing CNOT3 (green) expression in ZBTB16-positive (red) in undifferentiated spermatogonia with DAPI (blue).

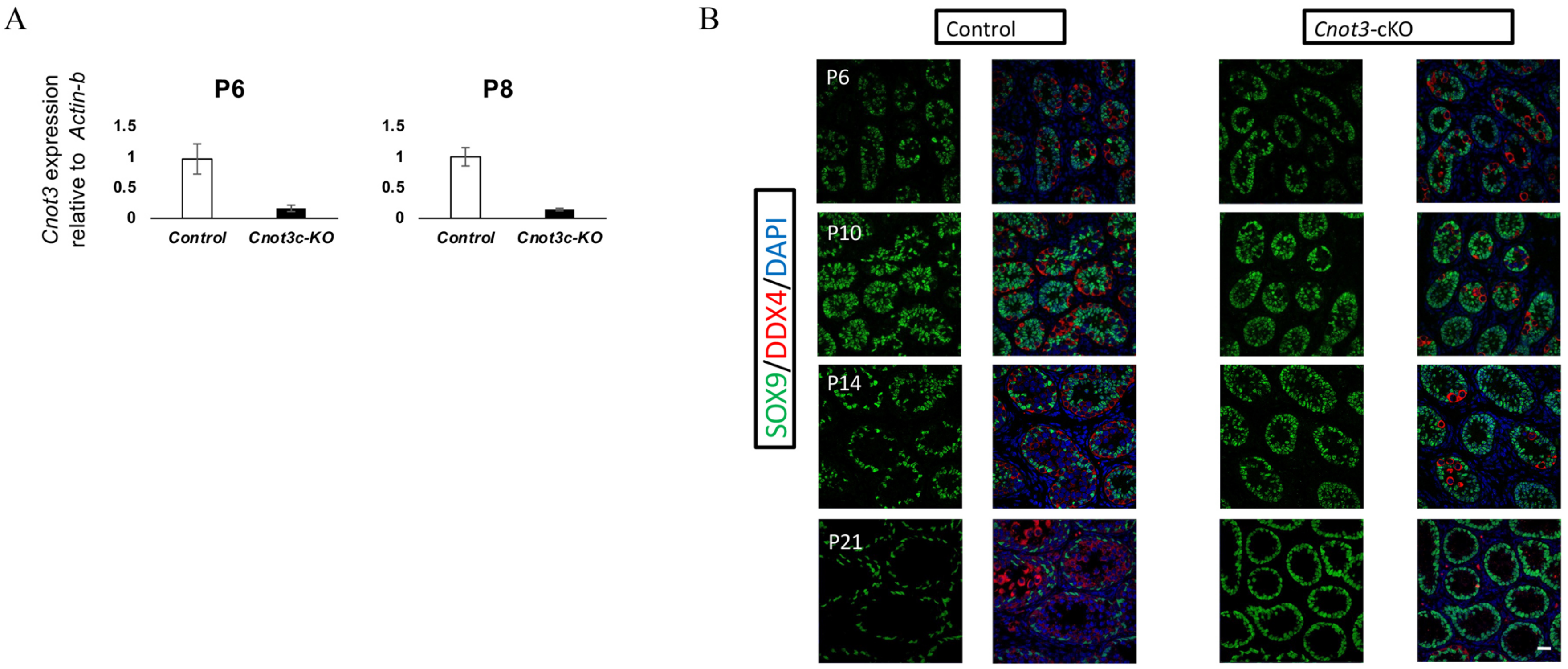

Fig. S2. Effect of *Cnot3* deletion on germ cell and Sertoli cells in juvenile testis

(A) Expression level of *Cnot3* relative to *β-actin* in SSCs Control and *Cnot3*-cKO ID4-EGFP+undifferentiation spermatogonia at P6 and P8. Mean ± SEM from three independent measurements are shown.

(B) Merged channels of single confocal sections of P6, P10, P14 and P21 Control and *Cnot3*-cKO testis showing SOX9 (green) and DDX4 (red) expression with DAPI (blue).

Development • Supplementary information

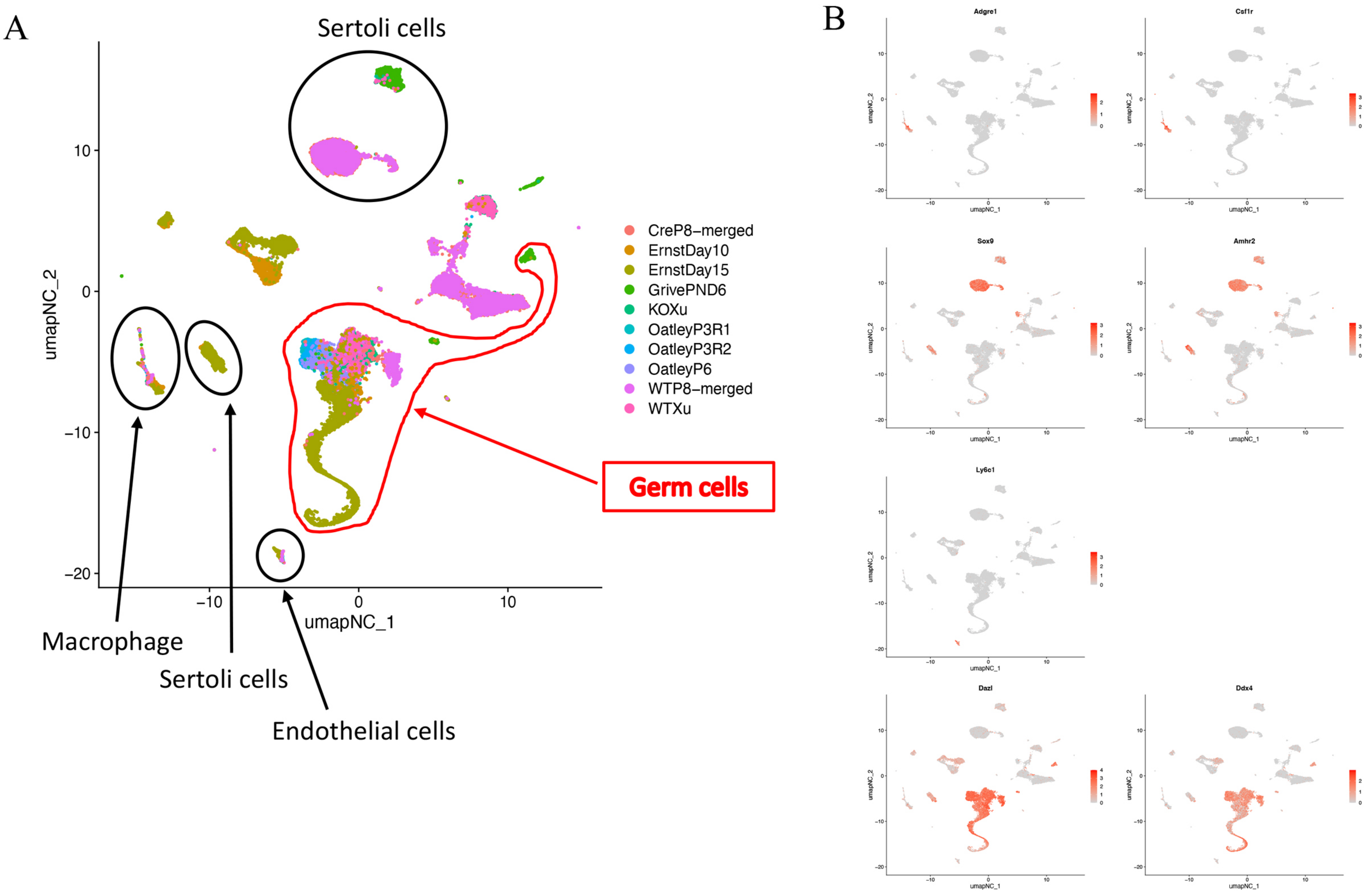

Fig. S3. Identification of the germ cells population based on clustering and marker gene expression

(A) UMAP projection of the testicular cells from the combination of our data with published datasets from P3, P6, P10, and P15 stages and indication of the Macrophage, Sertoli, Endothelial and germ cells in testicular (Law et al., 2019; Grive et al., 2019, Ernst et al, 2019).

(B) Highlights of of the Macrophage (Adgre1, Csf1r), Sertoli (Sox9, Amhr2), Endothelial (Lyc6c1) and germ cells (Dazl, Ddx4) in testicular in the UMAP projection of the testicular cells from the combination of our data with published datasets from P3, P6, P10, and P15 stages (Law et al., 2019; Grive et al., 2019, Ernst et al, 2019)

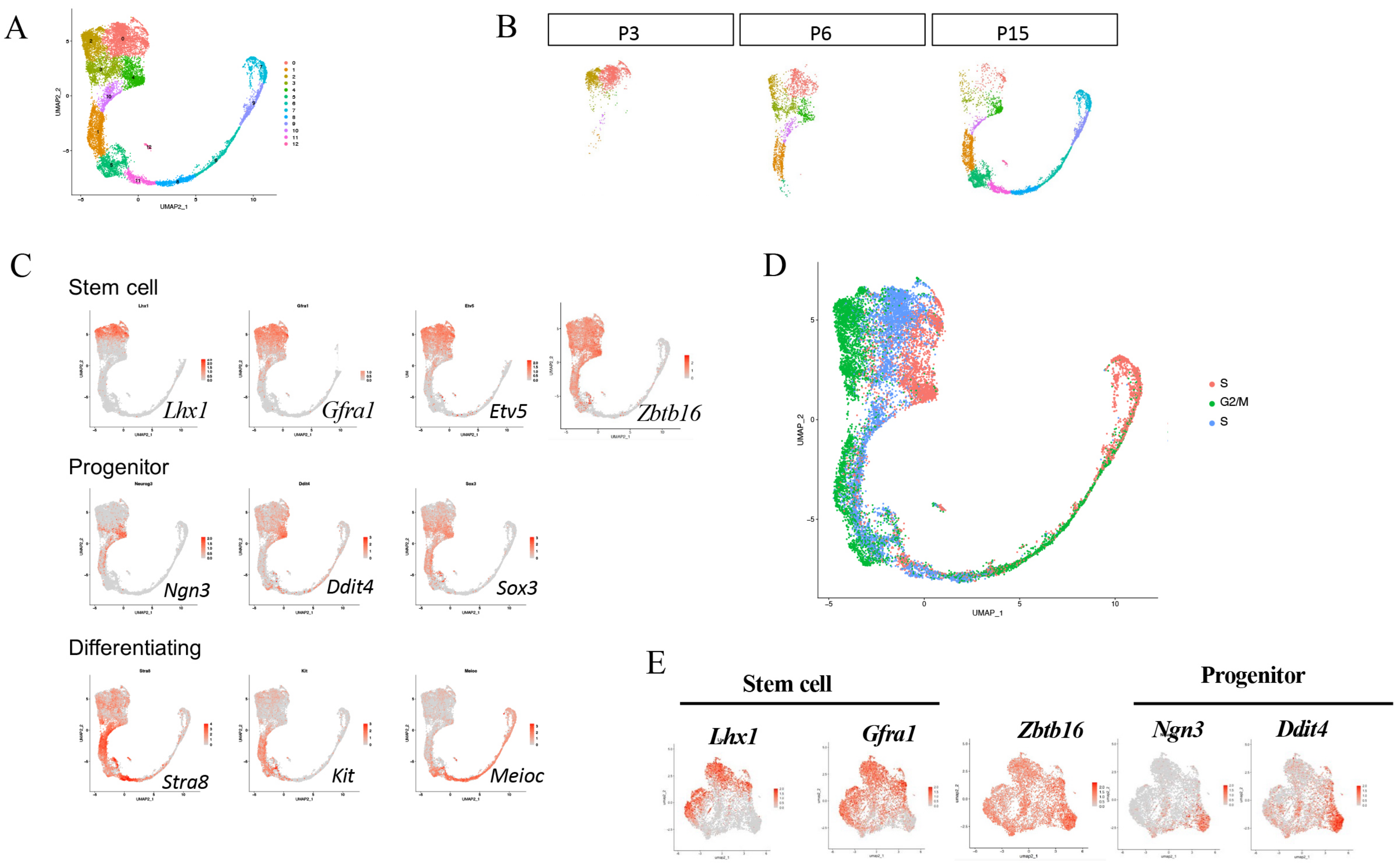

Fig. S4. Identification of the germ cells population based on clustering and marker gene expression

(A) Clustering of the of cells from the combination of our data with published datasets from P3, P6, and P15 stages (Law et al., 2019; Grive et al., 2019, Ernst et al, 2019).

(B) Clustering of the of cells from from the published datasets from P3, P6, and P15 stages, respectively (Law et al., 2019; Grive et al., 2019, Ernst et al, 2019).

(C) Highlights of Stem cells (*Lhx1, Gfra1 and, Etv5*), Progenitor cells (*Ngn3,Ddit4 and, Sox3*), differentiating (*Stra8 and c-Kit*) and maturing (Meioc) spermatogonia markers in clustered group of cells from the combination of our data with published datasets from P3, P6, P8, P10, and P15 stages (Law et al., 2019; Grive et al., 2019, Ernst et al, 2019).

(D) Highlights of cell cycle phases in clustered group of cells from the combination of our data with published datasets from P3, P6, P8, P10, and P15 stages (Law et al., 2019; Grive et al., 2019, Ernst et al, 2019).

(E) Highlights of Stem cells (*Lhx1* and *Gfra1*) and Progenitor cells (*Ngn3* and *Ddit4*) markers in reclustered group of cells containing SSCs and progenitor cells from the combination of our data with published datasets from P3, P6, P8, P10, and P15 stages (Law et al., 2019; Grive et al., 2019, Ernst et al, 2019).

Development • Supplementary information

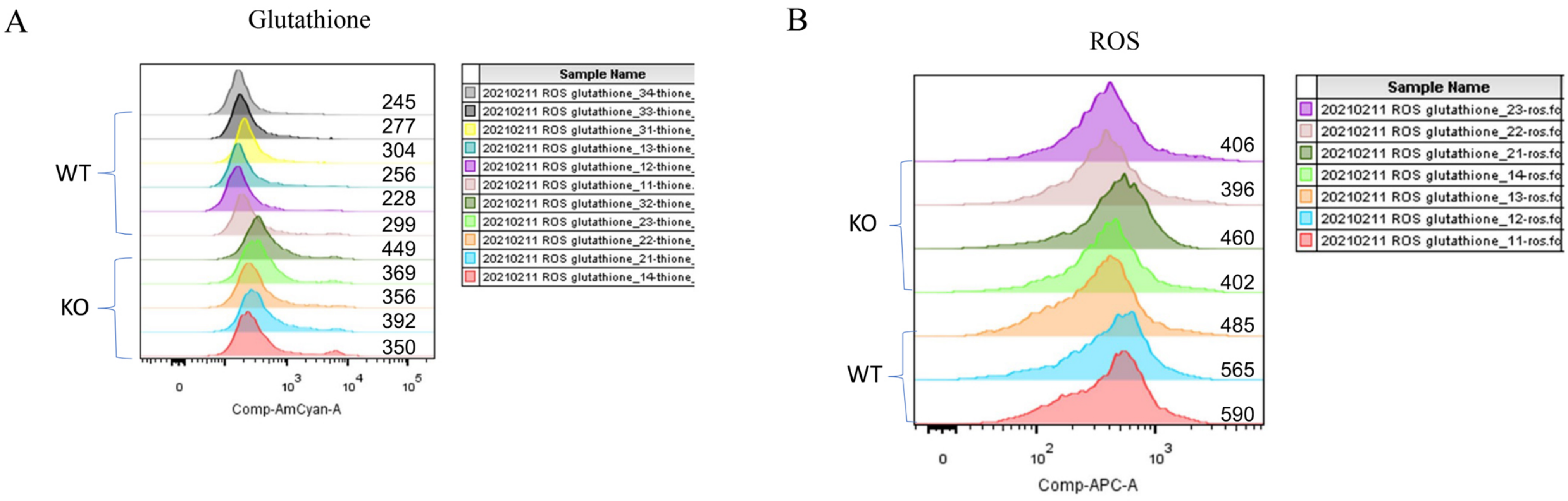

Fig. S5. Effect of *Cnot3* deletion on the glutathione and ROS content in ID4-EGFP+ undifferentiation spermatogonia at P8

(A) Glutathione content in Control and *Cnot3*-cKO ID4-EGFP+ undifferentiation spermatogonia at P8 analyzed by flow cytometry. An increase in Glutathione content resulted in an increase in the fluorescence intensity. Numbers indicate the median Glutathione signal in a.u.

(B) ROS content in content in Control and *Cnot3*-cKO ID4-EGFP+ undifferentiation spermatogonia at P8 analyzed by flow cytometry. A decrease in ROS content resulted in a decrease in the fluorescence intensity. Numbers indicate the median ROS signal in a.u.

Table S1. List of differentially expressed genes (DEGs) with a fold change >2 and adjusted P<0.01, when comparing RNA-seq data set from FACS sorted ID4-EGFP+ spermatogonia *Cnot3*-Control versus *Cnot3*-cKO. 798 transcripts were upregulated (DEGs_UP) and 137 were downregulated (DEGs-DOWN) in *Cnot3*-cKO ID4-EGFP+ spermatogonia as compared to controls.

Available for download at
https://journals.biologists.com/dev/article-lookup/doi/10.1242/dev.204557#supplementary-data

Table S2. List of Antibodies

Target	Application	Vendor	Dilution	Host
CNOT3	IF, WB	Proteintech (11135-1-AP)	1:200 (IF), 1:500 (WB)	Rabbit
CNOT3	IHC	Zheng et al., 2012	1:1000	Rabbit
CNOT1	WB	Proteintech (14276-1-AP)	1:500	Rabbit
CNOT2	WB	Proteintech (10313-1-AP)	1:3000	Rabbit
CNOT7	WB	Cell signaling (86665S)	1:1000	Rabbit
CNOT8	WB	Invitrogen (PA5-13451)	1:1000	Rabbit
β-Actin	WB	Sigma (A5441)	1:2000	Mouse
ZBTB16	IF	R&D (AF2944)	1:200	Goat
DDX4	IF	R&D (AF2030)	1:800	Goat
PCNA	IF	Santa Cruz (sc-7907)	1:200	Rabbit
c-CASP3	IF	CST(9661S)	1:300	Rabbit
SOX9	IF	Millipore(AB5535)	1:1000	Rabbit
STRA8	IF	Abcam (ab49602)	1:200	Rabbit
TRA98	IF	Abcam (ab82527)	1:1000	rat
c-KIT	IF	CST (3074)	1:400	Rabbit
GSTM1	IF	Proteintech (12412-1-AP)	1:200	Rabbit
anti-Rabbit IgG, Alexa Fluor™-488	IF	ThermoFisher (A-21206)	1:800	Donkey
anti-Goat IgG, Alexa Fluor™-594	IF	ThermoFisher (A-11058)	1:800	Donkey
anti-rat IgG, Alexa Fluor™-594	IF	ThermoFisher (**A-21209**)	1:800	Donkey
ECL anti-Rabbit IgG, HRP	WB	Amersham (NA934-V)	1:5000	Donkey

Development • Supplementary information

Table S3. Real time RT-PCR primers

Cnot1-F	GACTCGCTCTCGCTGGCCTTG
Cnot1-R	GCCTGTCTGCCTCAGGACCGTG
Cnot2-F	CCAACAGAAGCTCGCCAAGC
Cnot2-R	CCTGATTCCTGTTCATTCCAAATCCAG
Cnot3-F	AGAGGCCGATCTACAGATAGTGA
Cnot3-R	GACAGGCTTGGAGCCATTT
Gfra1-F	CACTCCTGGATTTGCTGATGT
Gfra1-R	AGTGTGCGGTACTTGGTGC
Zbtb16-F	GACGCACTACAGGGTTCACA
Zbtb16-R	GCTTGATCATGGCCGAGTAG
Sox9-F	ATCTGCACAACGCGGAGCTCA
Sox9-R	CTCTTCTCGCTCTCGTTCAGCAG
Gstm1-F	GCAGCTCATCATGCTCTGTT
Gstm1-R	TTTTCTCAGGGATGGTCTTCA
Gstm2-F	AGTTGGCCATGGTTTGCTAC
Gstm2-R	AGCTTCATCTTCTCAGGGAGAC
Gstm6-F	ATGGGCATGCTTTGCTACA
Gstm6-R	GGAACTCCGAGTAGAGTTTCAGC
Gstm7-F	GAGAGGAACCAAGTGTTTGAGGC
Gstm7-R	TTGGGAGGAAGCGACTGGTCTT
Gsta2-F	GAGCTTGATGCCAGCCTTCTGA

Development • Supplementary information

Gsta2-R	TTCTCTGGCTGCCAGGATGTAG
Gsta4-F	GATGATTGCCGTGGCTCCATTTA
Gsta4-R	CTGGTTGCCAACGAGAAAAGCC
MGST1-F	GCCAATCCAGAAGACTGTGTAGC
MGST1-R	AGGAGGCCAATTCCAAGAAATGG
MT1-F	ACCTCCTTGCAAGAAGAGCTGCT
MT1-R	GCTGGGTTGGTCCGATACTAT T
PTGES-F	TCCAGTATTACAGGAGTGACCCAG
PTGES-R	CCGAGGAAGAGGAAAGGATAGATT
Xdh-F	GCTCTTCGTGAGCACACAGAAC
Xdh-R	CCACCCATTCTTTTCACTCGGAC
bACTIN -F	TCCAGCCTTCCTTCTTGGGTAT
bACTIN -R	TCTTTACGGATGTCAACGTCACA

Development • Supplementary information

Supplemental Material and Method

Antibodies

Target	Application	Vendor	Host
CNOT3	IF, WB	Proteintech (11135-1-AP)	Rabbit
CNOT3	IHC	Zheng et al., 2012	Rabbit
CNOT1	WB	Proteintech (14276-1-AP)	Rabbit
CNOT2	WB	Proteintech (10313-1-AP)	Rabbit
CNOT7	WB	Cell signaling (86665S)	Rabbit
CNOT8	WB	Invitrogen (PA5-13451)	Rabbit
β-Actin	WB	Sigma (A5441)	
ZBTB16	IF	R&D (AF2944)	Goat
DDX4	IF	R&D (AF2030)	Goat
PCNA	IF	Santa Cruz (sc-7907)	Rabbit
c-CASP3	IF	CST(9661S)	Rabbit
SOX9	IF	Millipore(AB5535)	Rabbit
STRA8	IF	Abcam (ab49602)	Rabbit
TRA98	IF	Abcam (ab82527)	rat
c-KIT	IF	CST (3074)	Rabbit
GSTM1	IF	Proteintech (12412-1-AP)	Rabbit
anti-Rabbit IgG, Alexa Fluor™-488	IF	ThermoFisher (A-21206)	Donkey
anti-Goat IgG, Alexa Fluor™-594	IF	ThermoFisher (A-11058)	Donkey
anti-rat IgG, Alexa Fluor™-594	IF	ThermoFisher (**A-21209**)	Donkey
ECL anti-Rabbit IgG, HRP	WB	Amersham (NA934-V)	Donkey

Development • Supplementary information

Real time RT-PCR primers

Cnot1-F	GACTCGCTCTCGCTGGCCTTG
Cnot1-R	GCCTGTCTGCCTCAGGACCGTG
Cnot2-F	CCAACAGAAGCTCGCCAAGC
Cnot2-R	CCTGATTCCTGTTCATTCCAAATCCAG
Cnot3-F	AGAGGCCGATCTACAGATAGTGA
Cnot3-R	GACAGGCTTGGAGCCATTT
Gfra1-F	CACTCCTGGATTTGCTGATGT
Gfra1-R	AGTGTGCGGTACTTGGTGC
Zbtb16-F	GACGCACTACAGGGTTCACA
Zbtb16-R	GCTTGATCATGGCCGAGTAG
Sox9-F	ATCTGCACAACGCGGAGCTCA
Sox9-R	CTCTTCTCGCTCTCGTTCAGCAG
Gstm1-F	GCAGCTCATCATGCTCTGTT
Gstm1-R	TTTTCTCAGGGATGGTCTTCA
Gstm2-F	AGTTGGCCATGGTTTGCTAC
Gstm2-R	AGCTTCATCTTCTCAGGGAGAC
Gstm6-F	ATGGGCATGCTTTGCTACA
Gstm6-R	GGAACTCCGAGTAGAGTTTCAGC
Gstm7-F	GAGAGGAACCAAGTGTTTGAGGC
Gstm7-R	TTGGGAGGAAGCGACTGGTCTT

Gsta2-F	GAGCTTGATGCCAGCCTTCTGA
Gsta2-R	TTCTCTGGCTGCCAGGATGTAG
Gsta4-F	GATGATTGCCGTGGCTCCATTTA
Gsta4-R	CTGGTTGCCAACGAGAAAAGCC
MGST1-F	GCCAATCCAGAAGACTGTGTAGC
MGST1-R	AGGAGGCCAATTCCAAGAAATGG
MT1-F	ACCTCCTTGCAAGAAGAGCTGCT
MT1-R	GCTGGGTTGGTCCGATACTAT T
PTGES-F	TCCAGTATTACAGGAGTGACCCAG
PTGES-R	CCGAGGAAGAGGAAAGGATAGATT
Xdh-F	GCTCTTCGTGAGCACACAGAAC
Xdh-R	CCACCCATTCTTTTCACTCGGAC
bACTIN -F	TCCAGCCTTCCTTCTTGGGTAT
bACTIN -R	TCTTTACGGATGTCAACGTCACA
